# NMR-Based Metabolomics Analysis of Metabolite Profiles in Two Species of Boletes Subjected to Different Drying Methods

**DOI:** 10.3390/metabo15030152

**Published:** 2025-02-23

**Authors:** Yangzong Zhuoma, Minghong Yang, Yijie Chen, Xiangxi Zhang, Xingyan Duan, Hongwei Cui, Xin Fang, Xujia Hu

**Affiliations:** 1 Faculty of Life Science and Technology, Kunming University of Science and Technology, No. 727 South Jingming Road, Kunming 650500, China; 18760884008@163.com (Y.Z.); ymsh479@163.com (M.Y.); zxx990407ci@163.com (X.Z.); dxy2579@outlook.com (X.D.); 17390928042@163.com (H.C.); 2 College of Automotive Engineering, Jiangxi Polytechnic University, No. 1188, Shili Avenue, Lianxi District, Jiujiang 332007, China; sandychensunny@gmail.com; 3 Key Laboratory of Phytochemistry and Natural Medicines, Kunming Institute of Botany, Chinese Academy of Sciences, Kunming 650201, China

**Keywords:** boletes, drying methods, NMR, metabolomics

## Abstract

**Background:** Wild boletes are famous for their exceptional flavor and nutritional value. Due to their susceptibility to decay and spoilage, dry storage is a common method for processing and preservation. However, few studies have reported on the alterations of metabolites of boletes resulting from different drying methods. This paper aims to investigate the metabolic changes in two species of boletes, *Butyriboletus roseoflavus* and *Lanmaoa asiatica*, subjected to three drying methods: hot-air drying, microwave drying, and freeze drying. **Method and Result:** Nuclear magnetic resonance (NMR) metabolomics was utilized for multivariate data analysis. In total, 27 metabolites were identified from the two species of boletes, including amino acids such as glutamate and leucine, sugars like glucose and sucrose, and alkaloids like choline. Among these, 17 metabolites were classified as differential metabolites, comprising 12 amino acids, 4 sugars, and 1 alkaloid. Differential metabolites were quantified by quantitative NMR (qNMR), and these metabolites were subsequently analyzed using the Kyoto Encyclopedia of Genes and Genomes (KEGG) database for pathway enrichment analysis. KEGG pathway analysis revealed that the different drying methods resulted in significantly distinct metabolic pathways for these differential metabolites, resulting in the enrichment of amino acid metabolism and starch and sucrose metabolism pathways. **Conclusions:** This metabolomics study elucidates the differences in metabolite composition and abundance between the two species of boletes, providing a theoretical foundation for selecting appropriate drying methods for their preservation.

## 1. Introduction

Boletes are valuable edible mushrooms known for their unique flavor and medicinal properties. *Butyriboletus roseoflavus* has a cap diameter of 8–15 cm, which is flat to hemispherical in shape. The cap surface is smooth, firm, and thick. The cap surface and the base of the stem are rose-red. *Lanmaoa asiatica* has a cap diameter of 5–11 cm, which is hemispherical to broadly convex, sometimes wrinkled, and slightly incurved at the margin, shell pink, dull red to red; the cap is smooth and glossy [1]. They are rich in carbohydrates, proteins, amino acids, mineral ions, and vitamins [2]. Additionally, boletes contain bioactive compounds, including polyphenols, polysaccharides, and polypeptides, which exhibit antihypertensive, antitumor, antimicrobial, and immunomodulatory properties [3,4,5].

Boletes are seasonal delicacies that grow only in specific climates and seasons. Their high moisture content, rapid respiration rate, high enzyme activity, tender texture, and the presence of microorganisms make fresh boletes difficult to preserve for extended periods; it also makes them susceptible to damage and deterioration during storage and transportation [6,7]. Drying is the most effective preservation method for edible mushrooms, as it inhibits microbial growth, reduces enzyme activity, and slows adverse chemical reactions [8]. Currently, three primary drying methods are employed for fresh mushrooms: hot-air drying (HD), freeze drying (FD), and microwave drying (MD) [9]. HD employs electricity as an energy source to circulate hot air on the surface of the mushrooms, promoting the evaporation of moisture inside the mushrooms. Research indicates that HD may result in the loss of bioactive components due to prolonged drying times, resulting in decreased protein, polysaccharide, and fat content, but increased free amino acids content [10,11,12]. FD begins with the freezing of the material at low temperatures; the water is then removed in a vacuum by subliming ice into water vapor. The vacuum level directly impacts the sublimation rate and the preservation of metabolites. Inconsistent vacuum levels can lead to incomplete drying or damage to sensitive compounds. Standardizing vacuum settings and ensuring proper equipment calibration are essential [13]. FD retains most nutrients and microchemical composition, particularly preserving higher levels of polysaccharides and fats [14]. MD refers to the transfer of heat generated by microwave radiation to the boletes, causing the evaporation of water within them. MD may cause partial scorching due to uneven energy distribution and can also lead to a decrease in amino acids [15,16]. The secondary metabolite content of MD and FD is usually higher than that of HD [17]. Different drying methods yield different flavors. These methods convert proteins not only into amino acids but may also form peptides, accompanied by Maillard and enzymatic reactions [18,19]. In HD, appropriate temperatures can enhance enzyme activity and accelerate protein degradation to generate flavor compounds. Conversely, FD and MD may not be able to maintain appropriate temperatures, thereby reducing or inactivating enzyme activity [20]. Nevertheless, there are few studies on the drying methods of boletes, and even fewer on how different drying methods affect their chemical composition and content [21].

Although boletes are important edible mushrooms, the drying methods used are crucial for maintaining their quality and extending their shelf life. However, research on the drying methods for boletes remains relatively scant. Previous studies have predominantly focused on the effects of drying methods on specific metabolites, lacking a comprehensive analysis of the changes in various species of metabolites during the drying process [22]. Therefore, this study aims to explore in depth the variations in different metabolites throughout the drying process.

Metabolomics is commonly employed for the quantitative and qualitative analysis of metabolites [23]. It could establish a comprehensive metabolic profile that detects variations in biochemical composition across different drying methods while offering insights into the underlying causes of these changes [24]. Several advanced tools have been utilized in metabolomics, such as LC/MS [25,26], GC/MS [27], and UPLC/MS [28]. Nuclear magnetic resonance (NMR) is particularly advantageous for analyzing complex matrix systems, as it provides structural information and relative quantification of metabolites without the need for prior chemical separation or chromatography [29,30,31]. Due to these benefits, NMR is widely used for metabolite detection in various fields. Yang et al. [32] investigated flavor differences in stewed beef prepared with Chinese sauce using metabolomics and sensory evaluation. Pariyani et al. [33] studied the metabolite composition and antioxidant activity of *Orthosiphon stamineus* leaves by various drying methods. Additionally, NMR has also been employed to study the metabolomics of edible mushrooms [34,35,36,37,38]. However, few studies have been reported on the changes in the chemical composition of boletes under different drying methods.

To provide an appropriate processing method for wild boletes, this study combined NMR with metabolomics to investigate the metabolic changes in two species of boletes under three drying methods.

## 2. Materials and Methods

### 2.1. Plant Materials

Two species of fresh fruiting bodies, *B. roseoflavus* and *L. asiatica*, were purchased from the Mushuihua Wild Mushroom Trading Market in Kunming, Yunnan, China, in July 2023. They were identified by Prof. Zhuliang Yang from the Kunming Institute of Botany, Chinese Academy of Sciences (CAS). Samples were collected from mushrooms of consistent size.

### 2.2. Chemicals and Reagents

Methanol (analytical grade) was obtained from Aladdin Bio-Chem Technology Co., Ltd. (Shanghai, China). Deuterium oxide containing 99.9% D_2_O and 0.05% 3-(trimethylsilyl) propionic acid sodium salt was sourced from Sigma-Aldrich (St. Louis, MO, USA). Sodium hydroxide (NaOH, analytical grade) and monopotassium phosphate (KH_2_PO_4_, analytical grade) were acquired from Xilong Scientific Co., Ltd. (Sichuan, China). The reference standards were bought from the National Institutes for Food and Drug Control (Beijing, China) including leucine, isoleucine, valine, lactic acid, alanine, glutamate, methionine, malic acid, aspartate, asparagine, lysine, choline, arginine, taurine, glycine, threonine, mannitol, proline, *β*-glucose, *α*-glucose, trehalose, sucrose, adenosine, phenylalanine, and uridine.

### 2.3. Sample Preparation

Fresh and clean boletes were sliced into uniformly thin pieces and subjected to different drying methods to achieve a constant weight. After drying, the samples were ground into a fine powder and sealed in a dry dish.

Freeze Drying (FD): Dehydration was conducted at −55 °C with a vacuum degree of 1000 Pa for 24 h in a vacuum freeze dryer (FD-1A-50, Biocool, Beijing, China) after pre-freezing for 5 h in an ultra-low-temperature freezer (Homa, Guandong, China).

Hot-Air Drying (HD): The samples were placed on trays in an electric forced-air drying oven (GZX-9140MBE, Boxun, Shanghai, China). After drying at 30 °C for 5 h, they were further dried at 55 °C for 10 h.

Microwave Drying (MD): The samples were positioned on rotating trays within a microwave oven (M1-L202B, Midea, Guangdong, China). After an initial drying period of 3 min, intermittent drying continued for approximately 10 min. Microwave ovens operate at 700 W of microwave power.

### 2.4. Extraction Procedure

The extraction was conducted according to previous literature [39]. 500 mg of the samples were placed in 50 mL test tubes and sonicated with 10 mL of methanol–water (1:1, *v*/*v*) for 30 min at 25 °C. The materials were mixed in a gas bath thermostatic oscillator (Jtliangyou, Jiansu, China) at 25 °C for 30 min. After that, the materials were put into 50 mL centrifuge tubes, and they were centrifuged for 20 min at 10,000 rpm. The supernatant was treated with a rotary evaporator (Eyela, Beijing, China) at 37 °C to remove methanol. After that, the samples were pre-frozen for 5 h and then lyophilized in a vacuum freeze dryer to make sure that any remaining water was completely removed. The dried extracts were then stored at −80 °C for further processing.

The freeze-dried powder was dissolved in methanol and ultrapure water, respectively. The water solubility of freeze-dried powder is better than its methanol solubility; therefore, 10 mg of the freeze-dried powder was added to a 5 mL EP tube and dissolved in deuterium oxide (containing 99.9% D_2_O and 0.05% TSP; the pH of the D_2_O was adjusted to 6.8 using a phosphate buffer). Following this, the sample was vortexed for 1 min, and the mixture was centrifuged for 10 min at 10,000 rpm. A 600 µL sample was collected from the supernatant, filtered through 0.45 µm microfilters, and analyzed by NMR. Six independent extractions were performed for each drying method (*n* = 6).

### 2.5. NMR Spectroscopy Analysis and Data Processing

NMR spectra were measured using a 600 MHz Bruker Avance III HD Spectrometer (Bruker Biospin, Fällanden, Switzerland), operating at a frequency of 600.13 MHz at 25 °C. The following basic parameters were employed: number of points, 65,536; number of scans, 64; acquisition time, 3.26 s; spectral width, 20 ppm; and relaxation delay, 5 s. To suppress residual water peak signals, nuclear Overhauser effect spectroscopy (NOESY) pulses with presaturation were utilized. Deuterium oxide (D_2_O) served as an internal lock, while 0.05% TSP was used as an internal standard. The total measurement time was 2.6 min. All NMR spectra underwent phase adjustment and baseline correction and were compared to the internal standard (TSP) at 0.00 ppm using MestReNova 10.0. The water region (4.60–5.00 ppm) and the residual signal of methanol (3.35–3.38 ppm) were excluded from the raw data for analysis. Characteristic signals of metabolites (not fully overlapping) in the range of 0–10 ppm were integrated and normalized to the TSP signal. Metabolites of the boletes were identified and confirmed by comparing their peaks to data from the Human Metabolome Database (HMDB, http://www.hmdb.ca/, accessed on 1 December 2023), Biological Magnetic Resonance Data Bank (BMRB, https://bmrb.io/www/, accessed on 1 December 2023), and data from the literature [40,41,42].

### 2.6. Quantification of the Metabolites by NMR Method

NMR peaks are directly proportional to their molar concentrations [43]. The following equation can be used to quantify metabolites:

$$m_{X}=m_{ST}\times \frac{A_{x}}{A_{ST}}\times \frac{{MW}_{X}}{{MW}_{ST}}\times \frac{N_{ST}}{N_{X}}$$

where m_X_ is the unknown mass of the targeted analyte, and m_ST_ is the mass of the internal standard; A_X_ and A_ST_ are the integral areas for the selected signals; MW_X_ and MW_ST_ are the molecular weights of the targeted analyte and the standard; N_X_ and N_ST_ are the number of protons generating the integral signals, respectively.

### 2.7. Data Processing

Principal component analysis (PCA) and orthogonal projection to latent structure with discriminant analysis (OPLS-DA) were performed with SIMCA-P software (version 14.0, Umetrics, Umeå, Sweden) using unit variance scaling. To avoid overfitting, a validating supervised model with a 200X permutation test was used. To identify differential metabolites in boletes subjected to various drying methods, metabolites with variable importance in projection (VIP) value of ≥1 and a fold change of ≥2 (upregulated) or ≤0.5 (downregulated) were selected. The differential metabolites were illustrated in the volcano plot generated using Metware Cloud (https://cloud.metware.cn/, accessed on 10 January 2024). Hierarchical cluster analysis (HCA) involved normalizing metabolite content data using Z-score normalization and analyzing differential metabolites. Differential metabolites were annotated and utilized for pathway enrichment analysis through MetaboAnalyst (version 5.0; https://www.metaboanalyst.ca/, accessed on 10 January 2024).

## 3. Results

### 3.1. NMR Metabolite Analysis

**Figure 1:**
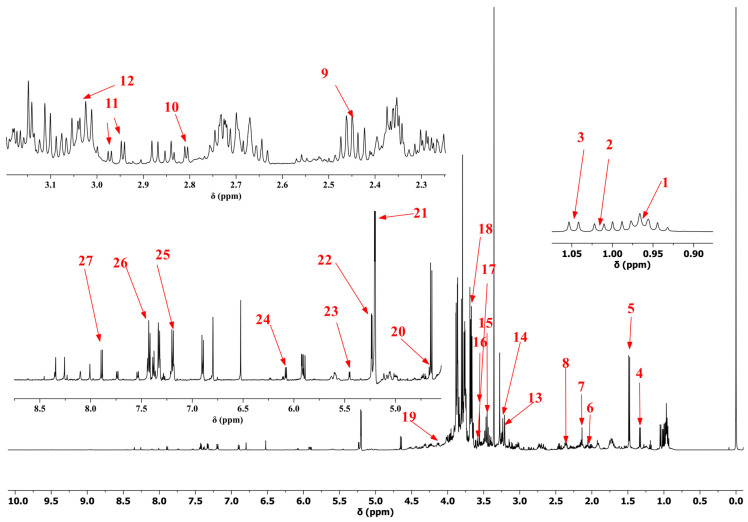
^1^H NMR spectra of extracts of bolete samples. Metabolites: (1) leucine, (2) isoleucine, (3) valine, (4) lactic acid, (5) alanine, (6) glutamate, (7) methionine, (8) malic acid, (9) glutamine, (10) aspartate, (11) asparagine, (12) lysine (13) choline, (14) arginine, (15) taurine, (16) glycine, (17) threonine, (18) mannitol, (19) proline, (20) *β*-glucose, (21) trehalose, (22) *α*-glucose, (23) sucrose, (24) adenosine, (25) tyrosine, (26) phenylalanine, and (27) uridine.

**Figure 2:**
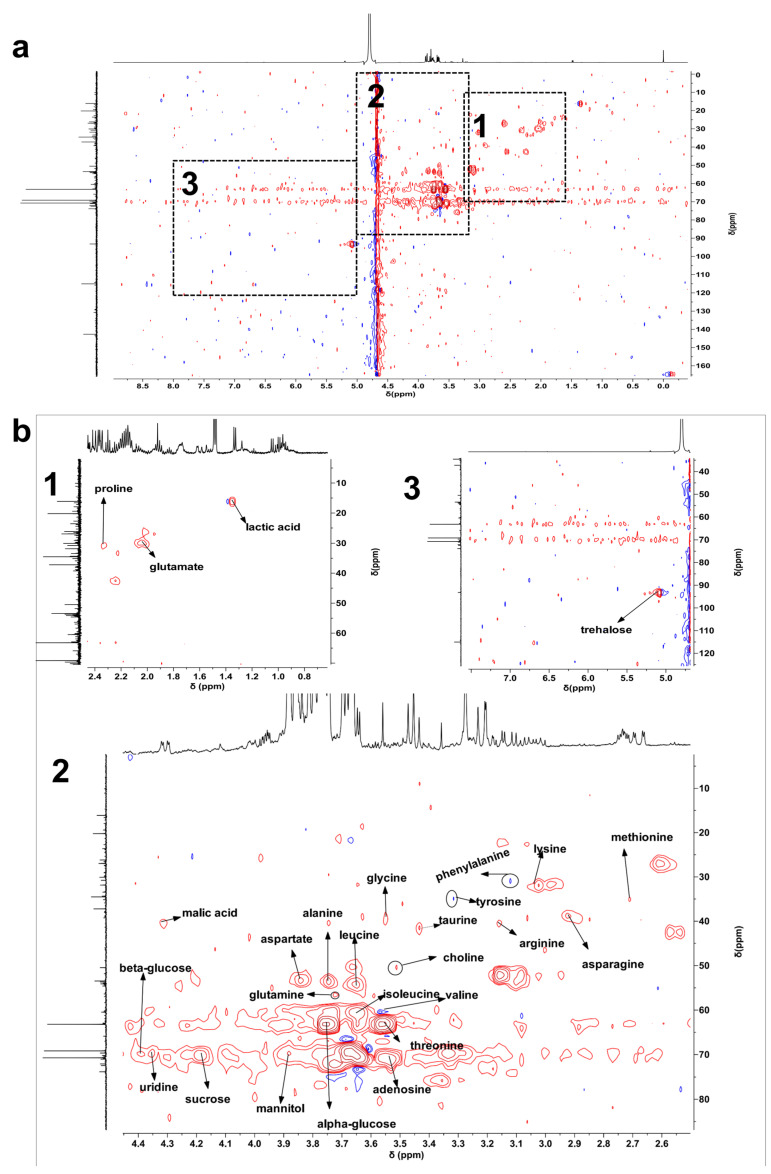
^1^H−^13^C HSQC spectrum of a representative bolete extract (**a**) and with an expanded region of interest used for metabolite assignments (**b**). Divide Figure 2a into three parts, labeled 1, 2, and 3, to correspond with the numbers in Figure 2b.

**Figure 3:**
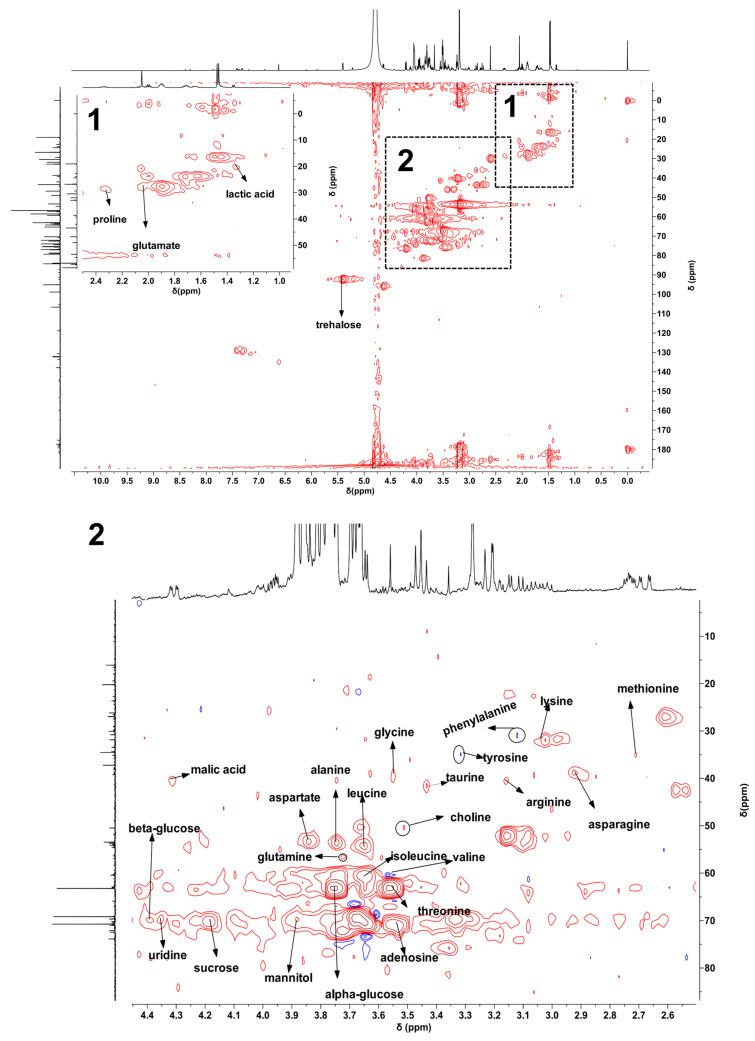
^1^H−^13^C HSQC spectrum of mixed standards with an expanded region of interest used for metabolite assignments. The 1 and 2 parts of the unlabeled metabolites were amplified to obtain the 1 and 2 parts of the labeled metabolites, respectively. The standards included leucine, isoleucine, valine, lactic acid, alanine, glutamate, methionine, aspartate, asparagine, lysine, choline, arginine, taurine, glycine, threonine, mannitol, proline, *β*-glucose, trehalose, *α*-glucose, sucrose, adenosine, phenylalanine, and uridine.

^1^H NMR spectra of bolete extracts from HD, FD, and MD samples are displayed in Figure 1 and Figure S1. The spectra revealed signals in the aliphatic (δ 0.8–3.0), carbohydrate (δ 3.0–5.5), and aromatic (δ 5.5–9.0) regions. However, some peaks overlapped in the ^1^H NMR spectra. To identify metabolites more accurately, we conducted two-dimensional map experiments (^1^H-^13^C HSQC) using sample and mixed standards (Figure 2 and Figure 3). Additionally, we performed two-dimensional map experiments (^1^H-^13^C HSQC) with individual standards for metabolites with significant overlap, including leucine, isoleucine, valine, lactic acid, threonine, methionine, and glutamate (Figure S2). A total of 27 metabolites were identified, including 17 amino acids (leucine, isoleucine, valine, threonine, alanine, glutamate, methionine, glutamine, aspartate, asparagine, lysine, arginine, taurine, glycine, proline, tyrosine, and phenylalanine), 2 organic acids (malic acid and lactic acid), 5 sugars (mannitol, *β*-glucose, trehalose, *α*-glucose, and sucrose), 1 alkaloid (choline) and 2 nucleotide metabolites (adenosine and uridine). Assignments for some signals are tabulated in Table S1. The spectral profiles obtained from the three drying methods reveal differences in the NMR signal intensity. The peak intensities of carbohydrate and aromatic regions varied in different samples, indicating that further research is needed to understand the difference between various drying methods.

### 3.2. Effect of Different Drying Methods on the Metabolite Profile of Boletes

**Figure 4:**
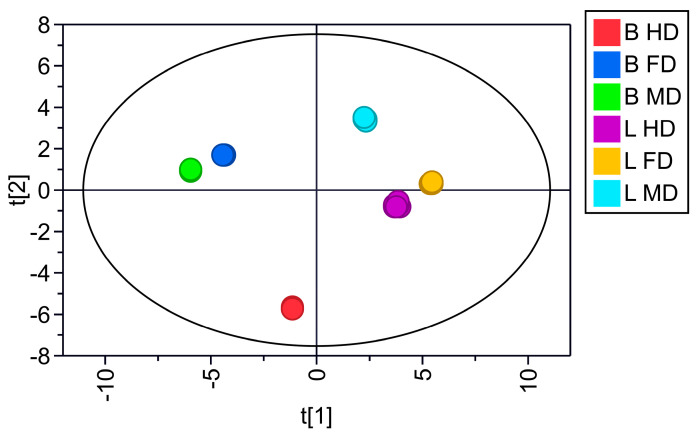
PCA score plot of three drying methods of *B. roseoflavus* and *L. asiatica*. t [1] and t [2] represent PC1 and PC2, respectively.

PCA was conducted to compare the metabolite profiles of two species of boletes subjected to the three drying methods. As shown in Figure 4, the first principal component (PC1) effectively distinguished the different groups of boletes well with a 49.1% variance contribution value, while the second principal component (PC2) distinguished the various drying methods based on a 24.8% variance contribution value. Six duplicate samples were collected using the same drying method, and no variations were observed among them, suggesting consistent components across all duplicates. By revealing significant differences in the composition of each dried sample, it indicates that under the influence of three different drying methods, the composition of the two bolete samples is significantly different.

### 3.3. Screening of Differential Metabolites in Boletes Treated with Different Drying Methods

OPLS-DA analysis is a multivariate statistical analysis method with supervised pattern recognition, used to screen for differential metabolites. Using OPLS-DA, the drying methods of *B. roseoflavus* and *L. asiatica* were analyzed in pairs, respectively.

**Figure 5:**
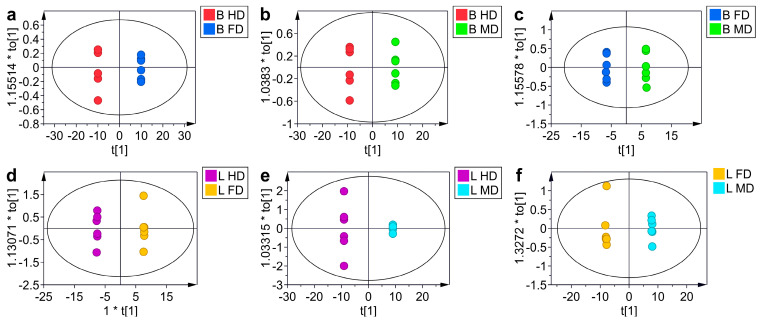
OPLS-DA of *B. roseoflavus* at freeze drying (BFD), hot-air drying (BHD), microwave drying (BMD), and *L. asiatica* at freeze drying (LFD), hot-air drying (LHD), microwave drying (LMD). (**a**–**f**) OPLS-DA model plots for the comparison groups BHD vs. BFD (R^2^Y = 1, Q^2^ = 1), BHD vs. BMD (R^2^Y = 1, Q^2^ = 1), BFD vs. BMD (R^2^Y = 0.999, Q^2^ = 0.999), LHD vs. LFD (R^2^Y = 0.999, Q^2^ = 0.998), LHD vs. LMD (R^2^Y = 1, Q^2^ = 0.999), and LFD vs. LMD (R^2^Y = 1, Q^2^ = 1).

The results, illustrated in Figure 5, demonstrated that the dried samples of each group were concentrated within the ellipse representing the 95% confidence interval. The dried samples were distributed on either side of the ellipse, indicating significant differences in the metabolites between the two groups. The R2Y and Q2 values of all established OPLS-DA models were greater than 0.99, indicating good model predictability and reproducibility [44]. Permutation tests also confirmed sufficient predictableness and goodness-of-fit (Figure S3). The blue Q2-values or green R2-values on the left were lower than the original points on the right, demonstrating the validity of the original model [45]. This approach further screens for differential metabolites that contribute significantly to pattern recognition.

**Figure 6:**
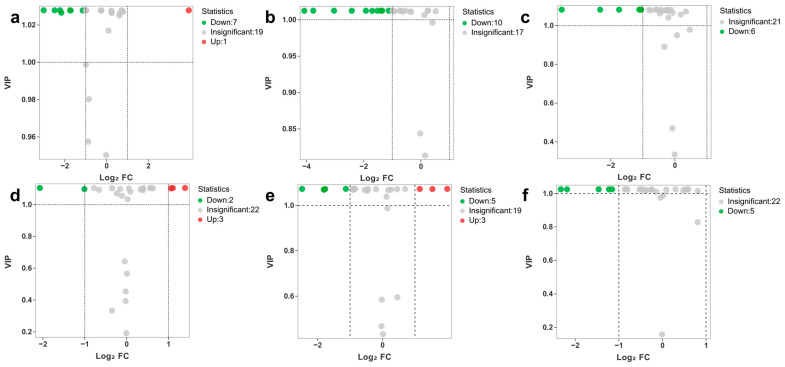
Volcano plots showing the differential metabolites. (**a**–**f**) Volcano plots for the comparison groups BHD vs. BFD, BHD vs. BMD, BFD vs. BMD, LHD vs. LFD, LHD vs. LMD, and LFD vs. LMD. A green spot indicates differentially expressed metabolites that have been downregulated; a red spot represents metabolites that have been upregulated; and a gray spot signifies those that have not significantly changed.

To identify differential metabolites, a fold change ≥2 (upregulated) or a fold change ≤0.5 (downregulated) and variable importance in projection (VIP) value ≥1 were established as screening criteria. Differential metabolites were displayed in the volcano plot (Figure 6). As shown in the results, 8 differential metabolites were identified (1 upregulated and 7 downregulated) in BHD when compared to BFD, 10 (10 downregulated) in BHD when compared to BMD, 6 (6 downregulated) in BFD when compared to BMD, 5 (3 upregulated and 2 downregulated) in LHD when compared to LFD, 8 (3 upregulated and 5 downregulated) in LHD when compared to LMD, and 5 (5 downregulated) in LFD when compared to LMD.

To provide a clearer understanding of individual metabolite levels across various dry methods, 17 differential metabolites were quantitatively analyzed using the qNMR method. The precision of the qNMR method was assessed through six replicate measurements of the same sample (BHD), yielding relative standard deviation (RSD) values ranging from 1.25% to 2.82%. The method repeatability was evaluated by analyzing six different working solutions independently prepared from the same sample (BHD) and the RSD values ranged from 1.36% to 3.78%. The stability of the same sample solution (BHD) was evaluated within 24 h and the RSD values ranged from 1.30 to 2.63%. Recovery tests were conducted to determine the accuracy of the qNMR method using the standard addition method, three different concentrations of each standard solution (low, medium, and high levels) were spiked into a known amount of the sample (BHD). The spiked samples were extracted, processed, and quantified according to the established methods and the average recoveries were between 91.06% and 106.36% with RSD values of less than 2.59% for 17 metabolites.

**Figure 7:**
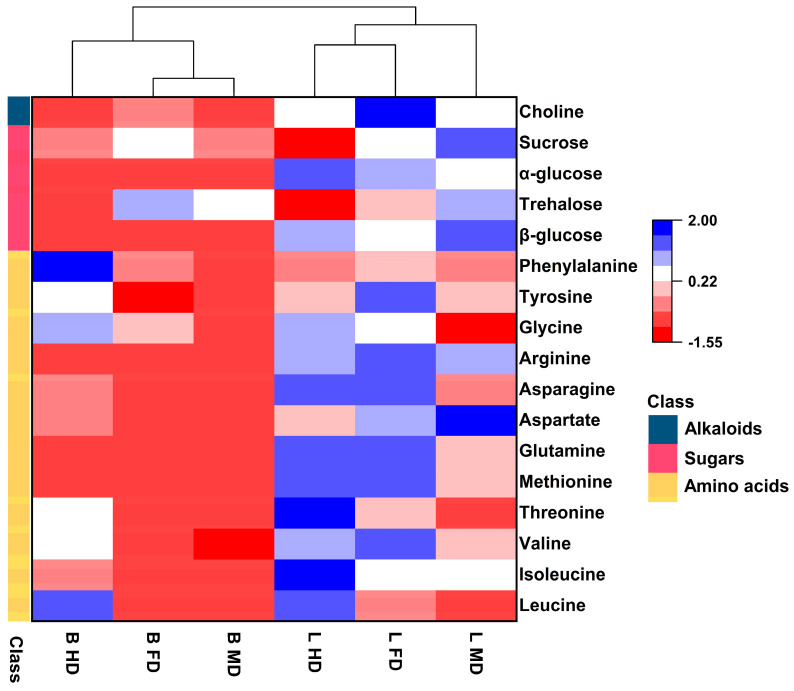
The clustered heat map of 17 differential metabolites at BHD vs. BFD, BHD vs. BMD, BFD vs. BMD, LHD vs. LFD, LHD vs. LMD, and LFD vs. LMD. Each metabolite is colored according to its accumulation level, from low (red) to high (blue).

To observe the variation patterns of the metabolites, the contents of six replicate metabolites for each treatment group were averaged and normalized. The quantified metabolites were plotted in a heat map (Figure 7). The result indicated that the two species of boletes were categorized into different groups. In total, 17 differential metabolites showed significant differences under different drying methods. Among them, FD and MD were clustered together in *B. roseoflavus*, while FD and HD were clustered together in L. asiatica, indicating that the metabolic profiles of different bolete samples were distinctly affected by drying methods. The 17 differential metabolites were classified into three groups: (1) 1 alkaloid, with higher content in FD than in MD and HD; (2) 4 types of sugars, with higher content in MD and FD than HD; (3) 12 types of amino acids, among which 9 amino acid contents in *B. roseoflavus* were higher in HD than in MD and FD, and 10 amino acid contents in *L. asiatica* were higher in HD and FD than in MD. The nutritional and flavor changes after drying may be linked to the differential metabolites, such as amino acids and sugars.

### 3.4. KEGG Pathway Analysis of Differential Metabolites

**Figure 8:**
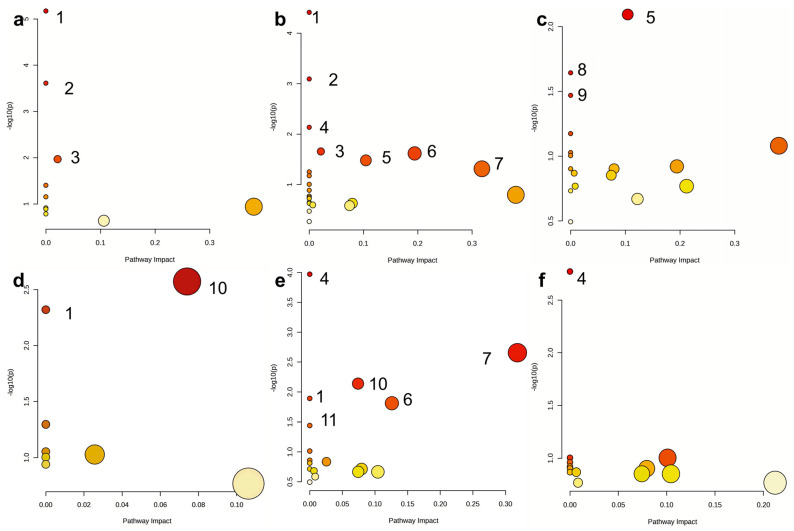
Metabolic pathway analysis. (**a**) BHD vs. BFD, (**b**) BHD vs. BMD, (**c**) BFD vs. BMD, (**d**) LHD vs. LFD, (**e**) LHD vs. LMD, (**f**) LFD vs. LMD. A bubble denotes an identified metabolic pathway. Colors of bubbles are proportional to pathway impact value (PIV), with red representing the highest level of significance (red) and white representing the lowest level of significance (white). Metabolic pathways: (1) valine, leucine and isoleucine biosynthesis, (2) valine, leucine and isoleucine degradation, (3) phenylalanine, tyrosine and tryptophan biosynthesis, (4) cyanoamino acid metabolism, (5) glyoxylate and dicarboxylate metabolism, (6) alanine, aspartate and glutamate metabolism, (7) glycine, serine and threonine metabolism, (8) biosynthesis of various plant secondary metabolites, (9) nitrogen metabolism, (10) starch and sucrose metabolism, (11) monobactam biosynthesis.

The identified metabolites were mapped onto the KEGG pathway to elucidate the compositional changes in boletes caused by different drying methods and to investigate the possible mechanisms affecting the metabolite content. According to KEGG pathway analysis results, the relative metabolic pathways are shown in Figure 8. The pathways were considered significantly enriched if the *p* values were less than 0.05, and the impact exceeded 0 [46].

In this study, we identified 10 metabolic pathways in BHD and BFD, including 3 major pathways (valine, leucine and isoleucine biosynthesis, valine, leucine and isoleucine degradation, and phenylalanine, tyrosine and tryptophan biosynthesis) as shown in Figure 8a (Table S2). Meanwhile, 23 metabolic pathways were identified between BHD and BMD, of which 7 major pathways (valine, leucine and isoleucine biosynthesis, valine, leucine and isoleucine degradation, phenylalanine, tyrosine and tryptophan biosynthesis, cyanoamino acid metabolism, glyoxylate and dicarboxylate metabolism, alanine, aspartate and glutamate metabolism, and glycine, serine and threonine metabolism) are presented in Figure 8b (Table S3). A total of 19 different metabolic pathways were identified between BFD and BMD, including 3 major metabolic pathways (glyoxylate and dicarboxylate metabolism, biosynthesis of various plant secondary metabolites and nitrogen metabolism) as shown in Figure 8c (Table S4). Compared with LHD, 8 and 19 metabolic pathways were identified in LFD and LMD, respectively, and 2 (valine, leucine and isoleucine biosynthesis and starch and sucrose metabolism) and 6 (valine, leucine and isoleucine biosynthesis, cyanoamino acid metabolism, alanine, aspartate and glutamate metabolism, glycine, serine and threonine metabolism, starch and sucrose metabolism and monobactam biosynthesis) metabolic pathways were significantly altered, as illustrated in Figure 8d,e (Tables S5 and S6). Overall, 15 metabolic pathways were identified in LFD and LMD, with cyanoamino acid metabolism being the major pathway, as shown in Figure 8f (Table S7). Additionally, some metabolic pathways overlapped within these comparative groups but were enriched at varying levels, indicating that different drying methods can significantly impact metabolite expression. These alterations in metabolic levels can deepen our comprehension of the effects of drying methods on boletes.

Valine, leucine and isoleucine metabolic pathways as well as phenylalanine, tyrosine and tryptophan biosynthesis were observed in BHD vs. BFD and BHD vs. BMD. Glyoxylate and dicarboxylate metabolism were identified in BHD vs. BMD and BFD vs. BMD. Valine, leucine and isoleucine biosynthesis, and starch and sucrose metabolism were commonly involved in LHD vs. LFD and LHD vs. LMD. Cyanoamino acid metabolism was widely affected in LHD vs. LMD and LFD vs. LMD. In this study, valine, leucine and isoleucine metabolic pathways, phenylalanine, tyrosine and tryptophan biosynthesis, and glyoxylate and dicarboxylate metabolism were identified as major pathways in *B. roseoflavus*. Valine, leucine and isoleucine biosynthesis, starch and sucrose metabolism as well as cyanoamino acid metabolism were major pathways in *L. asiatica*. These findings suggest that the metabolic pathways of different species of boletes vary under different drying methods. The determination of the metabolic pathway of boletes under different drying methods is closely linked to alterations in metabolites during processing. The corresponding pathways are illustrated in Figure S4.

## 4. Discussion

Metabolomics analysis revealed significant differences in the metabolites of boletes subjected to various drying methods, primarily involving amino acids, sugars, and alkaloids. Amino acids are the most prominent primary metabolites in edible mushrooms, significantly influencing their flavor, nutritional properties, and physiological activities [47,48]. As fundamental building blocks of proteins, amino acids play a crucial role in protein synthesis and are involved in regulating various physiological functions, including metabolic pathways and cell signaling. In addition to these roles, amino acids serve as the basic components of peptides, which directly affect the flavor and activity of mushrooms. For instance, umami peptides in mushrooms can impact the flavor [49]. Understanding the amino acid composition of mushrooms can enhance their nutritional value and promote their applications in the food industry. A study of these components can provide insights into processing for improved health benefits and experiences. In total, 12 types of amino acids were screened, including leucine, isoleucine, valine, threonine, methionine, glutamine, aspartic acid, asparagine, arginine, glycine, tyrosine, and phenylalanine, all of which are related to flavor, protein synthesis, and secondary metabolite production. In agreement with Yun et al. [50], the contents of most amino acids were found to be higher in HD compared to MD. This observation may be attributed to the decomposition of peptides and proteins at certain temperatures, which can yield a considerable proportion of free amino acids [51]. Leucine, isoleucine, valine, methionine, arginine, and phenylalanine are bitter amino acids, while glycine is recognized as a sweet amino acid [52]. In both species of boletes, the content of most bitter amino acids was lower in MD than in HD and FD, and glycine was higher in HD than in MD. Aspartic acid, known for its umami flavor, serves as a central carbon amino acid for synthesizing other amino acids. According to the study, the highest aspartic acid content was found in *L. asiatica* under HD treatment and in *B. roseoflavus* under MD treatment. This indicates that the expression of taste components in various boletes was differentially influenced by drying methods. Metabolic pathway analysis revealed that amino acid metabolism is significantly affected by the drying methods employed. The main metabolic pathways enriched in the comparisons between HD vs. FD and HD vs. MD groups included the biosynthesis and degradation of valine, leucine, and isoleucine, as well as the biosynthesis of phenylalanine, tyrosine, and tryptophan. The impact of HD on valine, leucine, and isoleucine metabolism was found to be greater than those of FD and MD. Branching-chain amino acids are involved in this metabolic pathway, which is primarily utilized for dietary treatments in conditions such as renal illness, muscular dystrophy, and chronic liver disease [53]. The biosynthesis of phenylalanine, tyrosine, and tryptophan involves aromatic amino acids, which perform various biological functions and serve as precursors for several phytohormones, including growth factors [54].

Sugars are the primary source of sweetness in mushrooms. This sweetness not only enhances the flavor of mushrooms but also increases their value in food processing applications. During the drying process, the evaporation of moisture leads to the concentration of sugars, making the flavor and texture of the dried mushrooms more pronounced. Additionally, sugars may undergo various physical and chemical changes during drying, such as the occurrence of the Maillard reaction, which can affect the color and flavor of the dried mushrooms, thereby further influencing consumer acceptance [55]. In this study, four differential sugar fractions were identified: *β*-glucose, trehalose, *α*-glucose, and sucrose. The contents of the four sugars in three different drying methods were different. Generally, sugar contents were higher in FD and HD samples compared to MD, likely due to high temperatures in the MD process, which can induce reactions such as the Maillard reaction, thereby affecting sugar contents [56]. Pathway analysis indicated significant enrichment in starch and sucrose metabolism for the comparisons between LHD vs. LFD and LHD vs. LMD. Notably, sucrose expression was significantly downregulated under HD treatment, potentially linked to its catabolism.

Alkaloids are a class of fundamental chemical compounds found in boletes, exhibiting a wide range of biological effects, including anti-inflammatory, antitumor, antifatigue, and antioxidant properties [37,57]. The content and bioactivity of alkaloids were significantly affected by drying. For example, excessive temperatures during drying may degrade sensitive alkaloids and reduce their efficacy. Only one differential alkaloid, choline, was identified through metabolomics analysis. Choline had the highest content observed under FD treatment, aligning with findings from Mohammad B. Hossain [58].

This study examined the impact of various drying techniques on the nutritional and sensory properties of boletes through a comprehensive metabolomics approach. While previous research has concentrated on nutritional composition or drying methods individually, there has been limited comparison of the alterations in nutritional content and flavor resulting from different drying techniques. Our findings hold significant implications for the drying and processing of seasonal fresh boletes.

## 5. Conclusions

In this study, NMR-based metabolomics was used to investigate the alterations in metabolites in two species of boletes (*B. roseoflavus* and *L. asiatica*) under three drying methods (HD, MD, and FD). A total of 27 types of metabolites were identified, revealing significant differences in their composition and concentration based on the drying technique applied. Volcano plots were generated to illustrate the differences in the upregulation and downregulation across the various drying treatments. Heat maps were also utilized to show the variations in the differential metabolites’ relative contents. The observed differences in the composition and concentration of amino acids, sugars, and alkaloids may underlie the variations in flavor and nutritional value of boletes under three different drying methods. KEGG pathway analysis revealed six critical metabolic pathways associated with the identified metabolites, suggesting that these methods alter not only the concentrations of metabolites but also the underlying biochemical pathways. This study provides valuable insights into the metabolic patterns influenced by different drying methods, enhancing our understanding of the changes in nutrients and flavor compounds in boletes. Moreover, further investigation is necessary to elucidate the mechanisms behind these molecular and metabolic alterations. Additionally, qualitative or quantitative analyses of volatile compounds and other factors that may affect flavor, and nutritional value should be considered.

## Data Availability

The data presented in this study are available upon request from the corresponding author.

## Supplementary Materials

The following supporting information can be downloaded at: https://www.mdpi.com/article/10.3390/metabo15030152/s1, Table S1. ^1^H NMR (δ) chemical shift, proton number, multiplicity of tentative metabolites identified in boletes. Table S2. BHD-BFD KEGG pathway analysis. Table S3. BHD-BMD KEGG pathway analysis. Table S4. BFD-BMD KEGG pathway analysis. Table S5. LHD-LFD KEGG pathway analysis. Table S6. LHD-LMD KEGG pathway analysis. Table S7. LFD-LMD KEGG pathway analysis. Figure S1. Metabolic profiles of *B.roseoflavus* at freeze drying (BFD), hot-air drying (BHD), microwave drying (BMD), and *L.asiatica* at freeze drying (LFD), hot-air drying (LHD), microwave drying (LMD). Figure S2. ^1^H−^13^C HSQC spectrum for the standards with peak overlap in the sample. The standards included leucine, isoleucine, valine, lactic acid, threonine, glutamate, and methionine. The standards were dissolved in the same buffer as the mushroom extract samples. Figure S3. 200X permutation test. Figure S4. Major KEGG pathway maps of identified metabolites of *B.roseoflavus* and *L.asiatica* at different drying methods, namely, (a) valine, leucine and isoleucine degradation, (b) phenylalanine, tyrosine and tryptophan biosynthesis, (c) valine, leucine and isoleucine degradation, (d) cyanoamino acid metabolism, (e) starch and sucrose metabolism, (f) glyoxylate and dicarboxylate metabolism.
