# Supplementary material for: NMR-Based Metabolomics Analysis of Metabolite Profiles in Two Species of Boletes Subjected to Different Drying Methods
Source: Metabolites. 2025 Feb 23;15(3):152. doi: 10.3390/metabo15030152 (PMC11943625; doi:10.3390/metabo15030152)
Supplement: Supplementary file 1 [file metabolites-15-00152-s001.zip › metabolites-3430353-supplementary.pdf]

Supplementary Materials

Table S1. ^{1}H NMR (δ) chemical shift, proton number, multiplicity of tentative metabolites identified in boletes

NO.	Metabolite	δ [1]H (ppm) and multiplicity#
1	Leucine	0.95(d), 0.97(d), 1.70(m), 1.74(m), 3.75(m)
2	Isoleucine	0.93(t), 1.02(d), 1.25(m), 1.97(m), 3.66(d)
3	Valine	0.99(d), 1.05(d), 2.27(m), 3.61(d)
4	Lactic acid	1.32(d), 4.11(q)
5	Alanine	1.47(d), 3.78(q)
6	Glutamate	2.04(m), 2.12(m), 2.33(m), 3.36(m), 3.75（m）
7	Methionine	2.11(m), 2.13(s), 2.19(m), 2.65(t), 3.85(dd)
8	Malic acid	2.36(dd), 2.66(dd), 4.30(dd)
9	Glutamine	2.13(m),2.44(m), 3.76(t)
10	Aspartate	2.70(dd), 2.80(dd), 3.89(dd)
11	Asparagine	2.85(dd), 2.94(dd) 4.00(dd),
12	Lysine	1.45(m), 1.71(m), 1.91(m), 3.02(t), 3.75(t)
13	Choline	3.19(s), 3.51(m), 4.06(m)
14	Arginine	1.64(m), 1.72(m), 1.89(m), 3.22(t), 3.76(t)
15	Taurine	3.25(t), 3.44(t)
16	Glycine	3.54(s)
17	Threonine	1.32(d), 3.58(d), 4.25(m)
18	Mannitol	3.67(dd), 3.75(m), 3.79(d), 3.86(dd)
19	proline	1.97(m), 2.02(m), 2.06(m), 2.34(m), 3.33(m), 3.41(m), 4.12(dd)
20	β-Glucose	3.25(dd), 3.48(dd), 3.89(dd), 4.64(d)
21	Trehalose	3.44(t), 3.64(dd), 3.76(dd), 3.81(m), 5.18(d)
22	α-Glucose	3.53(dd), 3.71(m), 3.82(m), 3.85(dd), 5.22(d),
23	Sucrose	3.46(t), 3.55(dd), 3.66(d), 3.68(d), 3,75(t), 3.79(dd), 3.81(dd), 3.82(m),3.88(m), 4.02(t), 4.21(d), 5.44(d),
24	Adensodine	3.83(dd), 3.90(dd), 4.28(m), 4.42(q), 4.79(t), 6.08(d)
25	Tyrosine	3.04(dd), 3.19(dd), 6.89(d), 7.18(d),
26	Phenylalanine	3.12(dd), 3.28(dd), 3.99(dd), 7.31(m), 7.37(m), 7.42(m)
27	Uridine	3.79(dd), 3,90(dd), 4.12(m), 4.21(t), 4.34(t), 5.88(d), 5.90((d), 7.88(d)

Table S2. BHD-BFD KEGG pathway analysis.

pathway	total	expected	hits	Raw p	Holm adjust	FDR
Valine, leucine and isoleucine biosynthesis	20	0.16018	4	6.72E-06	0.00049036	0.00049036
Valine, leucine and isoleucine degradation	18	0.14416	3	0.0002445	0.017604	0.0089243
Phenylalanine, tyrosine and tryptophan biosynthesis	21	0.16819	2	0.010746	0.76294	0.26148
Phenylalanine metabolism	5	0.040046	1	0.0519498	1	0.72085

Tyrosine metabolism	9	0.072082	1	0.070127	1	1
Starch and sucrose metabolism	15	0.12014	1	0.1145	1	1
Ubiquinone and other terpenoid-quinone biosynthesis	16	0.12815	1	0.12172	1	1
Galactose metabolism	17	0.13616	1	0.12888	1	1
Pantothenate and CoA biosynthesis	22	0.1762	1	0.16396	1	1
Glycine, serine and threonine metabolism	32	0.25629	1	0.2305	1	1

Table S3. BHD-BMD KEGG pathway analysis.

pathway	total	expected	hits	Raw p	Holm adjust	FDR
Valine, leucine and isoleucine biosynthesis	20	0.22883	4	3.85E-05	0.0028141	0.0028141
Valine, leucine and isoleucine degradation	18	0.20595	3	0.00080622	0.058048	0.029427
Cyanoamino acid metabolism	12	0.1373	2	0.0073216	0.51983	0.17816
Phenylalanine, tyrosine and tryptophan biosynthesis	21	0.24027	2	0.022043	1	0.35185
Alanine, aspartate and glutamate metabolism	22	0.25172	2	0.024099	1	0.35185
Glyoxylate and dicarboxylate metabolism	26	0.29748	2	0.033083	1	0.40251
Glycine, serine and threonine metabolism	32	0.36613	2	0.048662	1	0.50748
Phenylalanine metabolism	5	0.057208	1	0.056039	1	0.51136
Nitrogen metabolism	6	0.06865	1	0.066902	1	0.54265
Tyrosine metabolism	9	0.10297	1	0.098818	1	0.72137
Vitamin B6 metabolism	12	0.1373	1	0.12975	1	0.86106
Starch and sucrose metabolism	15	0.17162	1	0.15972	1	0.86122
Ubiquinone and other terpenoid-quinone biosynthesis	16	0.18307	1	0.1695	1	0.86122
Galactose metabolism	17	0.19451	1	0.17918	1	0.86122
Arginine biosynthesis	18	0.20595	1	0.18876	1	0.86122
Thiamine metabolism	18	0.20595	1	0.18876	1	0.86122
Pantothenate and CoA biosynthesis	22	0.25172	1	0.22607	1	0.9035
Porphyrin metabolism	23	0.26316	1	0.23516	1	0.9035
Methane metabolism	23	0.26316	1	0.23516	1	0.9035
Lipoic acid metabolism	25	0.28604	1	0.25304	1	0.91019
Glutathione metabolism	26	0.29748	1	0.26183	1	0.91019
Pyrimidine metabolism	35	0.40046	1	0.33692	1	1
Purine metabolism	65	0.74371	1	0.54021	1	1

Table S4. BFD-BMD KEGG pathway analysis.

pathway	total	expected	hits	Raw p	Holm adjust	FDR
Glyoxylate and dicarboxylate metabolism	26	0.14874	2	0.0080593	0.58833	0.58833
Biosynthesis of various plant secondary metabolites	4	0.022883	1	0.022726	1	0.73237
Nitrogen metabolism	6	0.034325	1	0.033934	1	0.73237
Cyanoamino acid metabolism	12	0.06865	1	0.06694	1	0.73237
Vitamin B6 metabolism	12	0.06865	1	0.06694	1	0.73237
Starch and sucrose metabolism	15	0.085812	1	0.083101	1	0.73237
Galactose metabolism	17	0.097254	1	0.09375	1	0.73237
Arginine biosynthesis	18	0.10297	1	0.099037	1	0.73237
Thiamine metabolism	18	0.10297	1	0.099037	1	0.73237
Alanine, aspartate and glutamate metabolism	22	0.12586	1	0.11994	1	0.73237
Porphyrin metabolism	23	0.13158	1	0.12511	1	0.73237

Methane metabolism	23	0.13158	1	0.12511	1	0.73237
Lipoic acid metabolism	25	0.14302	1	0.13536	1	0.73237
Glutathione metabolism	26	0.14874	1	0.14045	1	0.73237
Glycerophospholipid metabolism	32	0.18307	1	0.1705	1	0.77793
Glycine, serine and threonine metabolism	32	0.18307	1	0.1705	1	0.77793
Pyrimidine metabolism	35	0.20023	1	0.18521	1	0.79532
Cysteine and methionine metabolism	41	0.23455	1	0.214	1	0.86789
Purine metabolism	65	0.37185	1	0.32113	1	1

Table S5. LHD-LFD KEGG pathway analysis.

pathway	total	expected	hits	Raw p	Holm adjust	FDR
Starch and sucrose metabolism	15	0.085812	2	0.0026711	0.19499	0.17439
Valine, leucine and isoleucine biosynthesis	20	0.11442	2	0.0047777	0.344	0.17439
Tyrosine metabolism	9	0.051487	1	0.050551	1	1
Ubiquinone and other terpenoid-quinone biosynthesis	16	0.091533	1	0.088438	1	1
Galactose metabolism	17	0.097254	1	0.09375	1	1
Valine, leucine and isoleucine degradation	18	0.10297	1	0.099037	1	1
Phenylalanine, tyrosine and tryptophan biosynthesis	21	0.12014	1	0.11475	1	1
Glycine, serine and threonine metabolism	32	0.18307	1	0.1705	1	1

Table S6. LHD-LMD KEGG pathway analysis.

Pathway	total	expected	hits	Raw p	Holm adjust	FDR
Cyanoamino acid metabolism	12	0.10984	3	0.00010686	0.0078007	0.0078007
Glycine, serine and threonine metabolism	32	0.29291	3	0.0022078	0.15896	0.080584
Starch and sucrose metabolism	15	0.1373	2	0.0072586	0.51536	0.17662
Valine, leucine and isoleucine biosynthesis	20	0.18307	2	0.012835	0.89844	0.22573
Alanine, aspartate and glutamate metabolism	22	0.20137	2	0.015461	1	0.22573
Monobactam biosynthesis	4	0.036613	1	0.036175	1	0.44013
beta-Alanine metabolism	11	0.10069	1	0.096732	1	0.92497
Lysine biosynthesis	16	0.14645	1	0.13792	1	0.92497
Galactose metabolism	17	0.15561	1	0.14596	1	0.92497
Arginine biosynthesis	18	0.16476	1	0.15393	1	0.92497
Valine, leucine and isoleucine degradation	18	0.16476	1	0.15393	1	0.92497
Thiamine metabolism	18	0.16476	1	0.15393	1	0.92497
Porphyrin metabolism	23	0.21053	1	0.19283	1	0.92497
Methane metabolism	23	0.21053	1	0.19283	1	0.92497
Lipoic acid metabolism	25	0.22883	1	0.20794	1	0.92497
Glutathione metabolism	26	0.23799	1	0.2154	1	0.92497
Glyoxylate and dicarboxylate metabolism	26	0.23799	1	0.2154	1	0.92497
Glycerophospholipid metabolism	32	0.29291	1	0.25891	1	1
Cysteine and methionine metabolism	41	0.37529	1	0.3202	1	1

Table S7. LFD-LMD KEGG pathway analysis.

pathway	total	expected	hits	Raw p	Holm adjust	FDR
Cyanoamino acid metabolism	12	0.06865	2	0.0016906	0.12342	0.12342

Valine, leucine and isoleucine degradation	18	0.10297	1	0.099037	1	0.7887
Thiamine metabolism	18	0.10297	1	0.099037	1	0.7887
Arginine biosynthesis	18	0.10297	1	0.099037	1	0.7887
Valine, leucine and isoleucine biosynthesis	20	0.11442	1	0.10954	1	0.7887
Alanine, aspartate and glutamate metabolism	22	0.12586	1	0.11994	1	0.7887
Pantothenate and CoA biosynthesis	22	0.12586	1	0.11994	1	0.7887
Porphyrin metabolism	23	0.13158	1	0.12511	1	0.7887
Methane metabolism	23	0.13158	1	0.12511	1	0.7887
Arginine and proline metabolism	25	0.14302	1	0.13536	1	0.7887
Lipoic acid metabolism	25	0.14302	1	0.13536	1	0.7887
Glutathione metabolism	26	0.14874	1	0.14045	1	0.7887
Glyoxylate and dicarboxylate metabolism	26	0.14874	1	0.14045	1	0.7887
Glycerophospholipid metabolism	32	0.18307	1	0.1705	1	0.82979
Glycine, serine and threonine metabolism	32	0.18307	1	0.1705	1	0.82979

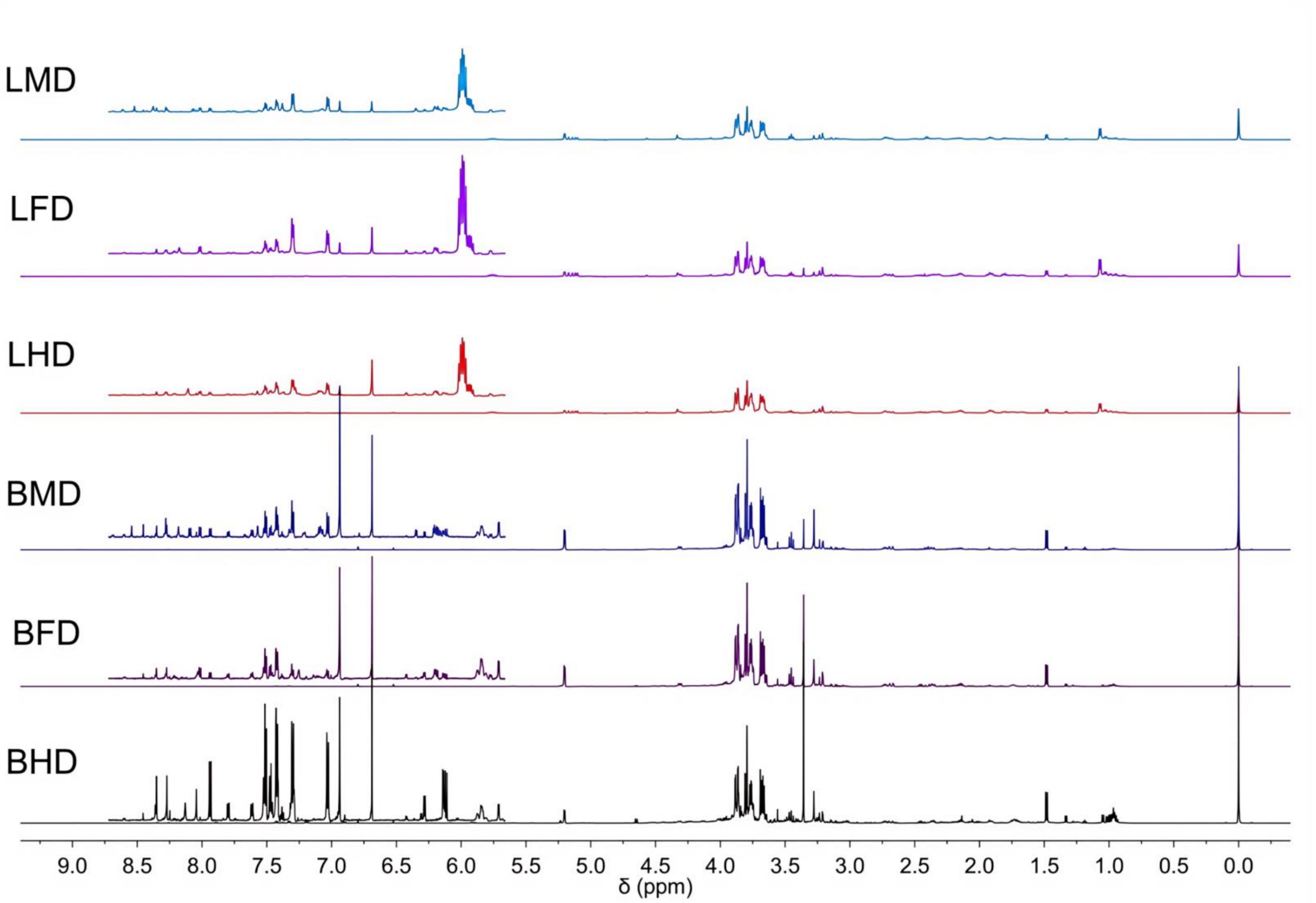

Figure S1. Metabolic profiles of *B. roseoflavus* at freeze drying (BFD), hot-air drying (BHD), microwave drying (BMD), and *L. asiatica* at freeze drying (LFD), hot-air drying (LHD), microwave drying (LMD).

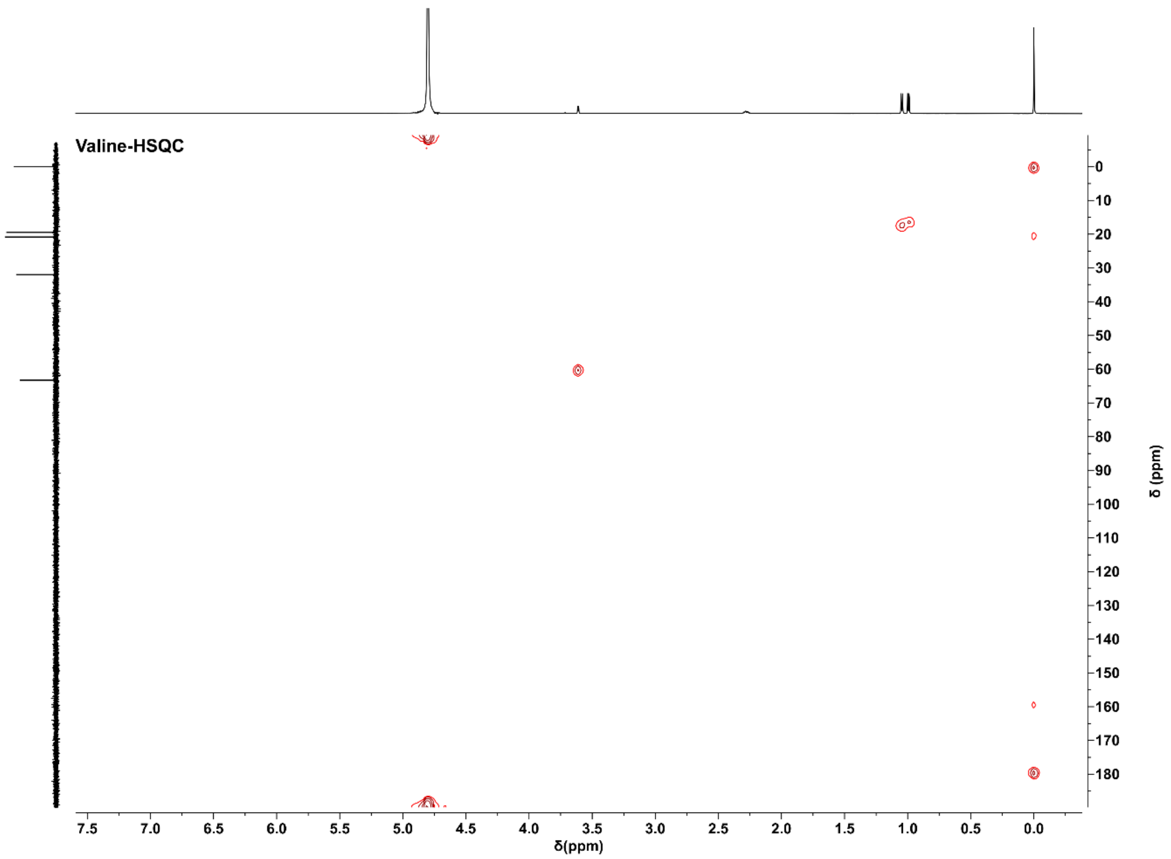
Valine-HSQC
δ(ppm)
δ (ppm)
7.5
7.0
6.5
6.0
5.5
5.0
4.5
4.0
3.5
3.0
2.5
2.0
1.5
1.0
0.5
0.0
0
10
20
30
40
50
60
70
80
90
100
110
120
130
140
150
160
170
180

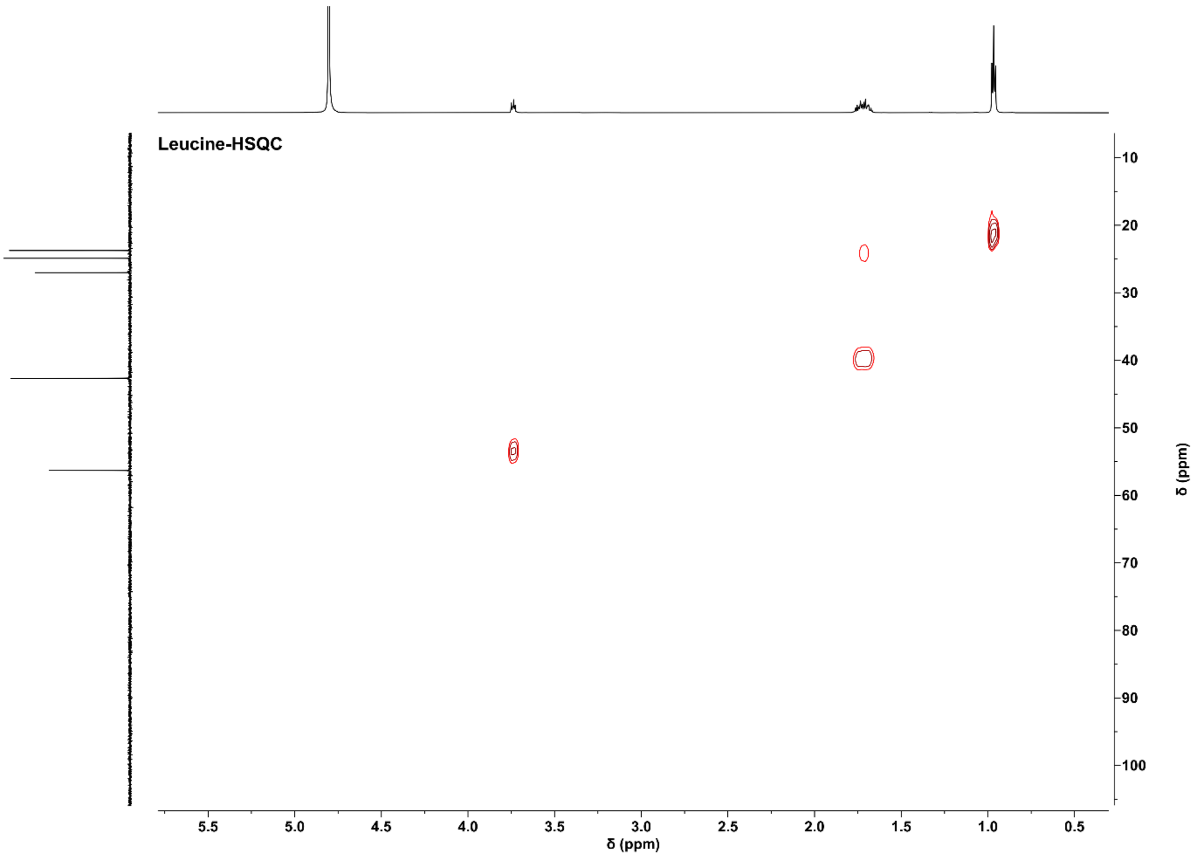
Leucine-HSQC
δ (ppm)
δ (ppm)
5.5
5.0
4.5
4.0
3.5
3.0
2.5
2.0
1.5
1.0
0.5
10
20
30
40
50
60
70
80
90
100

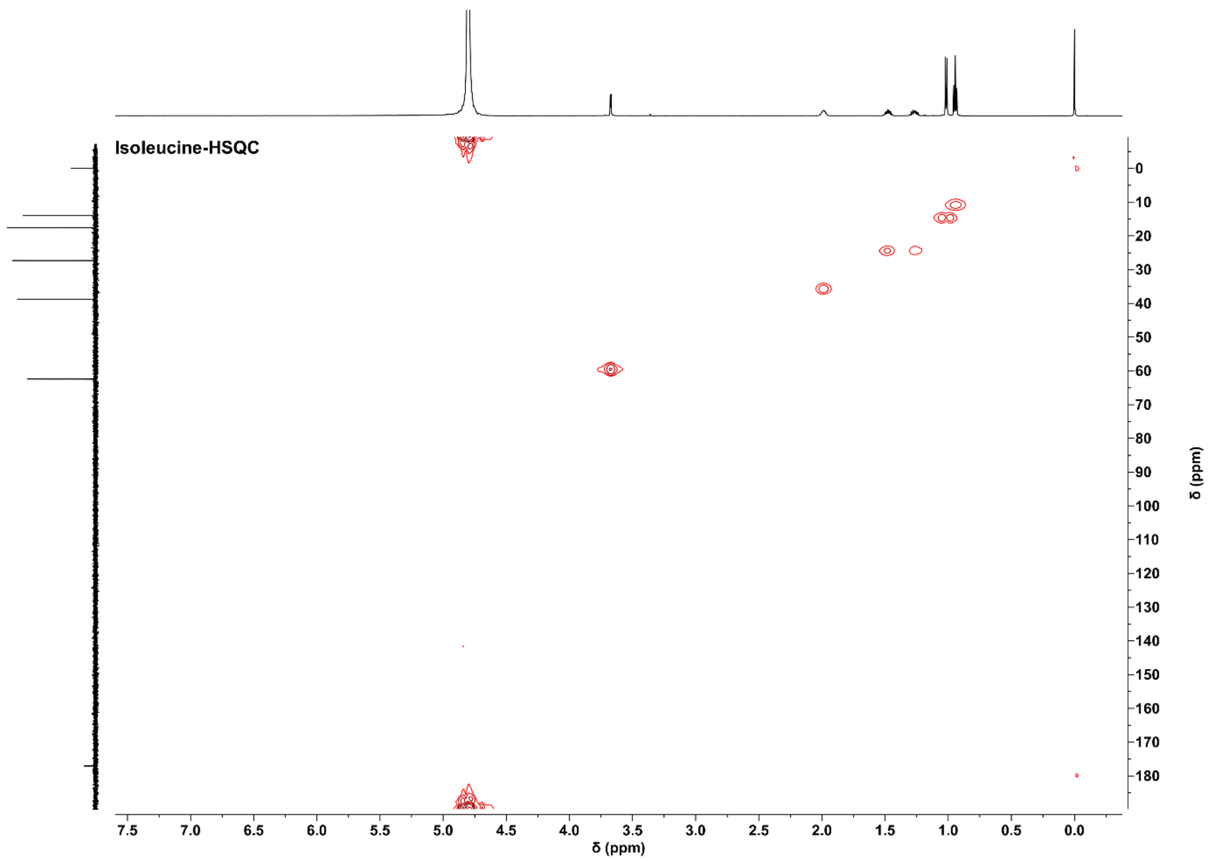
Isoleucine-HSQC
δ (ppm)
δ (ppm)

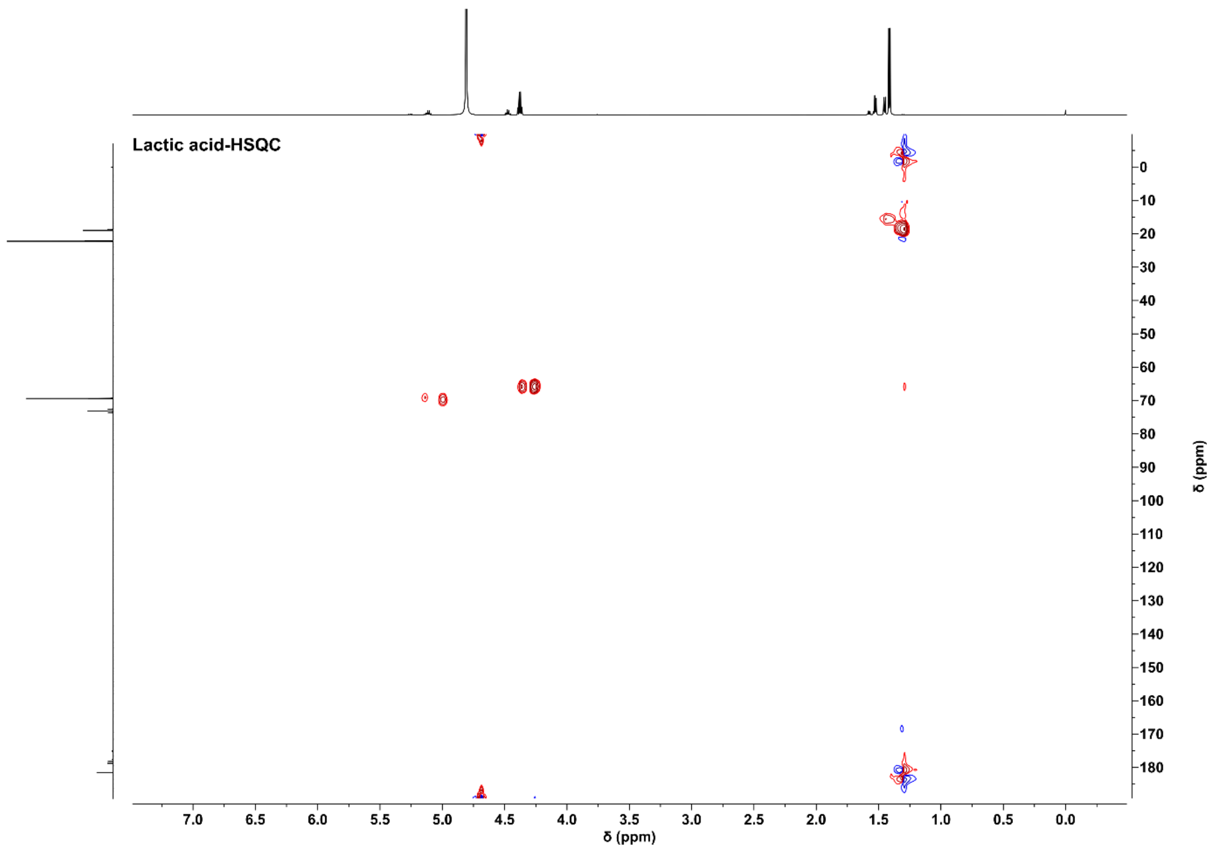
Lactic acid-HSQC
δ (ppm)
δ (ppm)

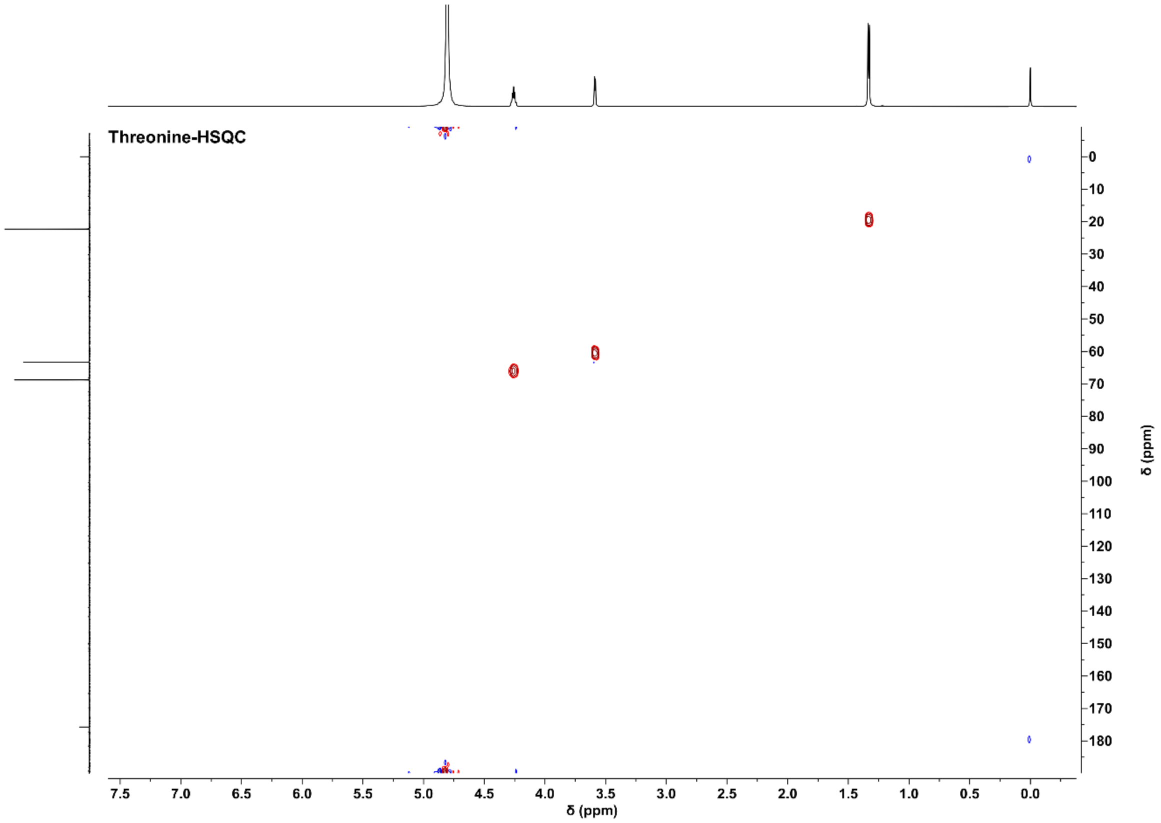
Threonine-HSQC
δ (ppm)
δ (ppm)

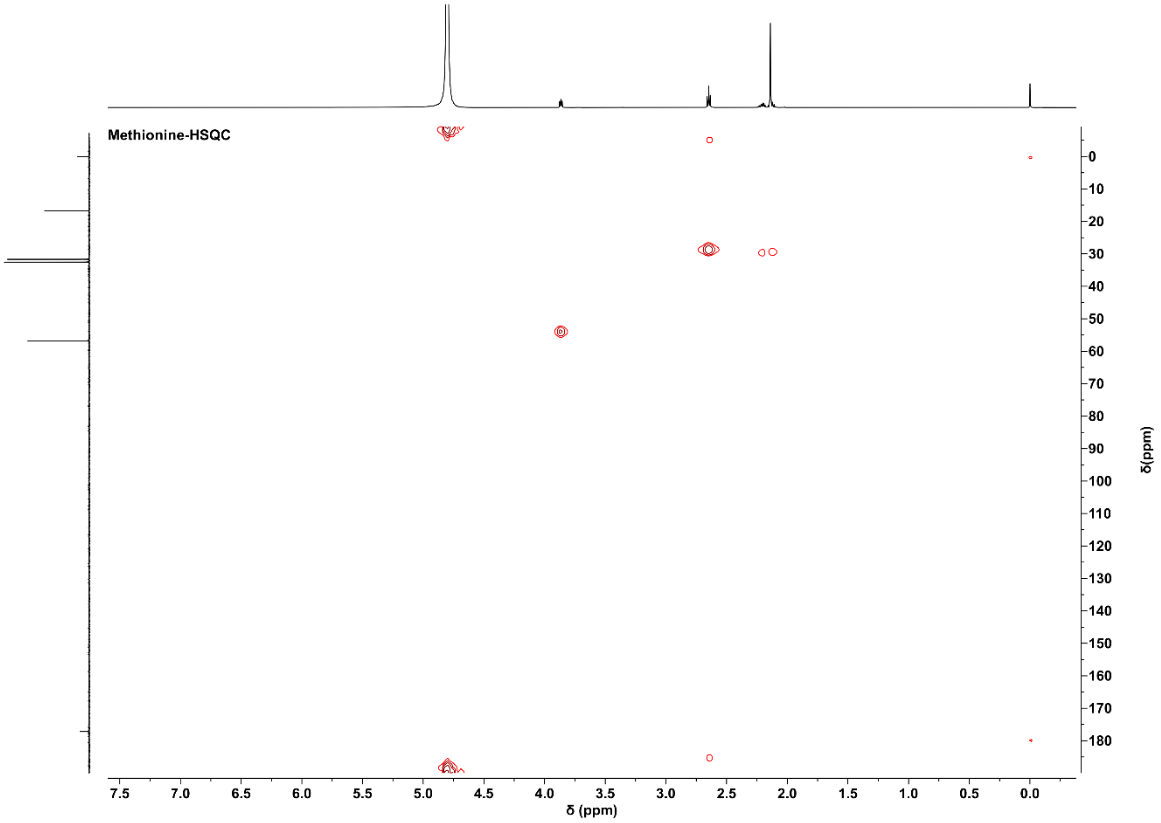
Methionine-HSQC
δ (ppm)
δ(ppm)

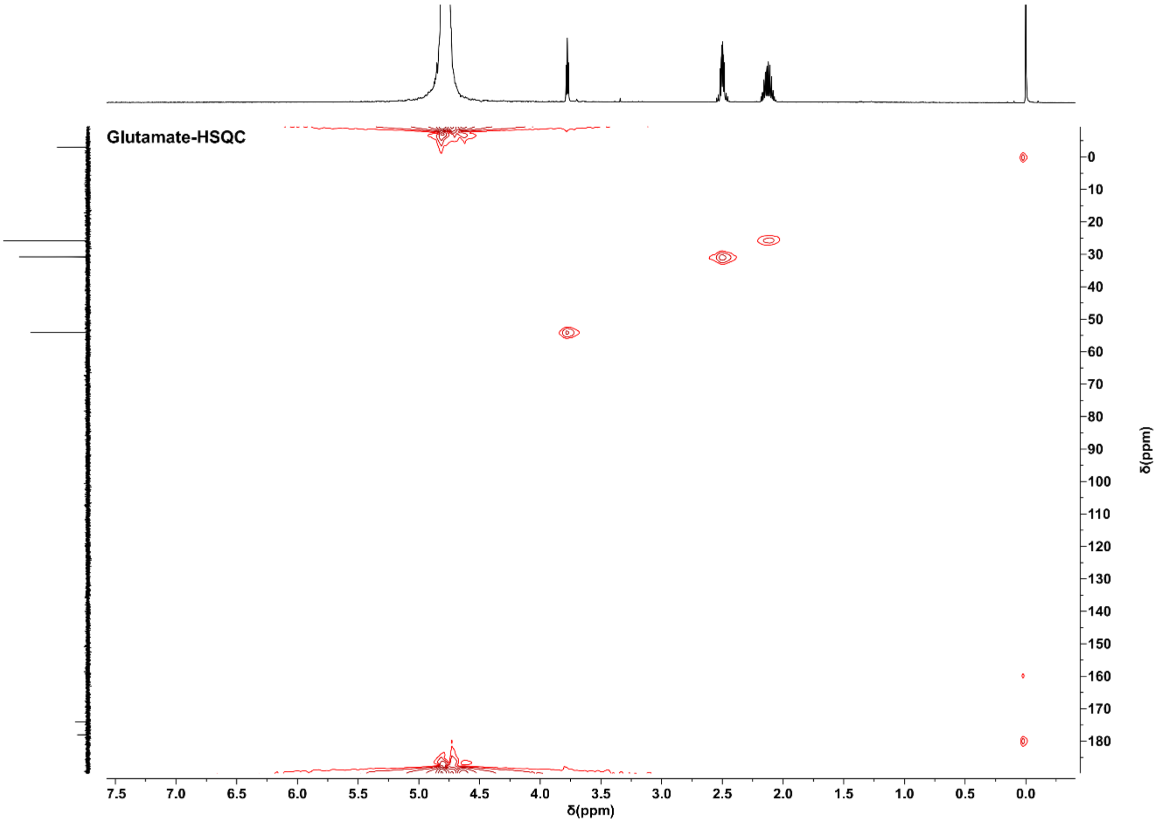

Figure S2. ^{1}H–^{13}C HSQC spectrum for the standards with peak overlap in the sample. The standards included leucine, isoleucine, valine, lactic acid, threonine, glutamate, and methionine. The standards were dissolved in the same buffer as the mushroom extract samples.

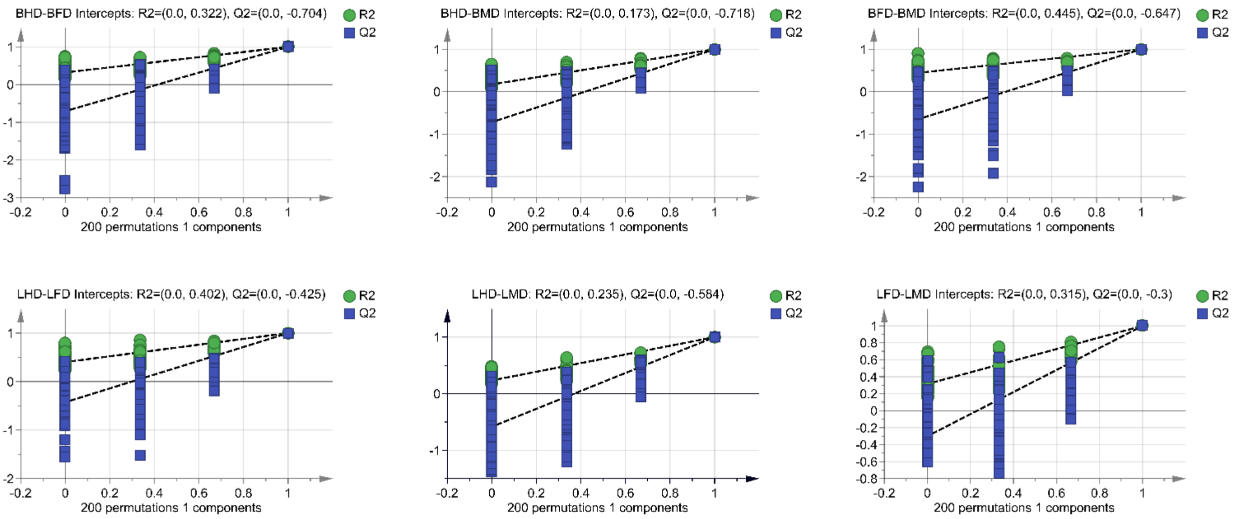

Figure S3. 200X permutation test.

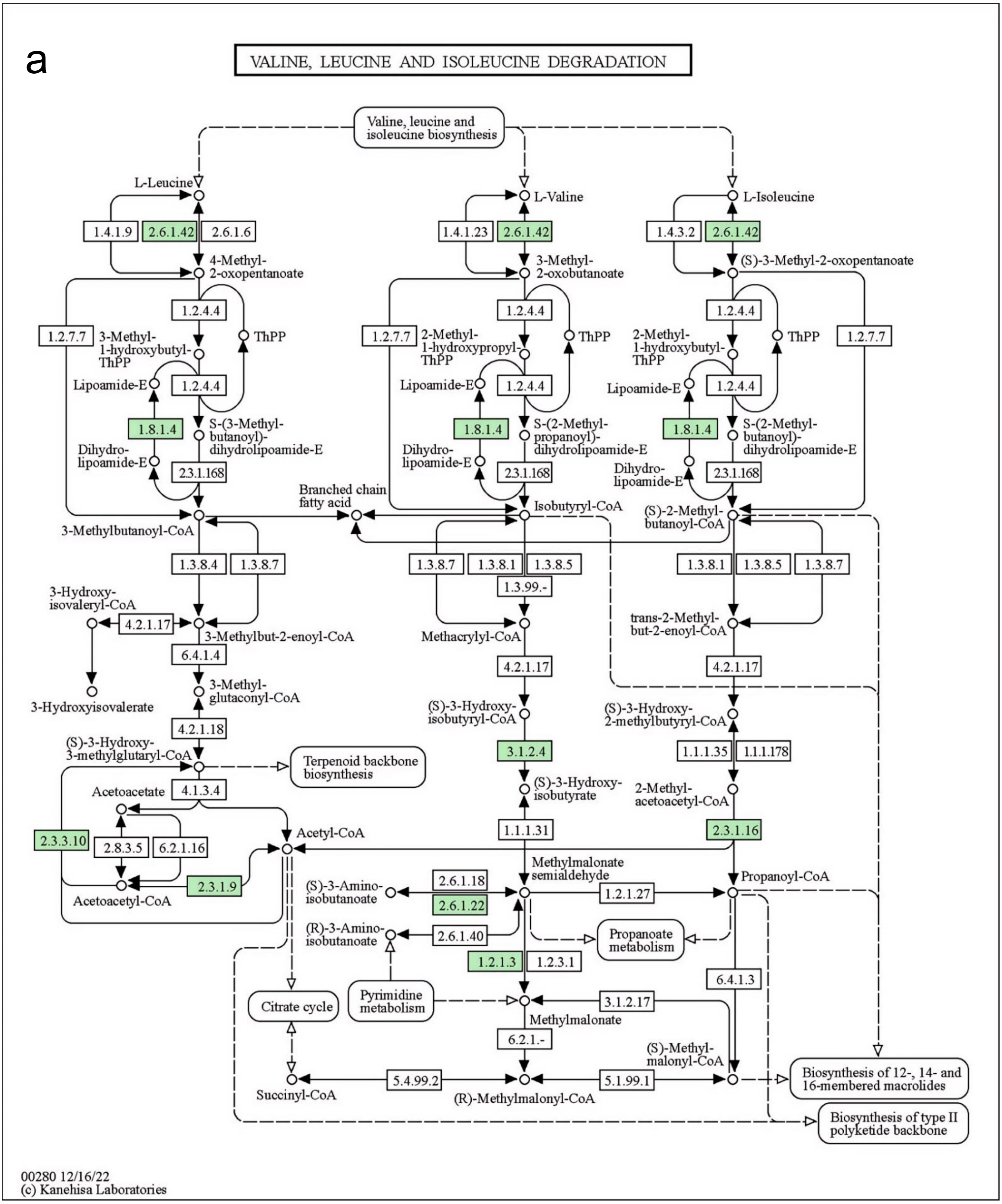
a
VALINE, LEUCINE AND ISOLEUCINE DEGRADATION
Valine, leucine and isoleucine biosynthesis
L-Leucine
L-Valine
L-Isoleucine
1.4.1.9
2.6.1.42
2.6.1.6
1.4.1.23
2.6.1.42
1.4.3.2
2.6.1.42
4-Methyl-2-oxopentanoate
3-Methyl-2-oxobutanoate
(S)-3-Methyl-2-oxopentanoate
1.2.4.4
1.2.7.7
ThPP
3-Methyl-1-hydroxybutyl-ThPP
2-Methyl-1-hydroxypropyl-ThPP
2-Methyl-1-hydroxybutyl-ThPP
Lipoamide-E
1.8.1.4
Dihydro-lipoamide-E
S-(3-Methylbutanoyl)-dihydrolipoamide-E
S-(2-Methylpropanoyl)-dihydrolipoamide-E
S-(2-Methylbutanoyl)-dihydrolipoamide-E
2.3.1.168
Branched chain fatty acid
3-Methylbutanoyl-CoA
Isobutyryl-CoA
(S)-2-Methylbutanoyl-CoA
1.3.8.4
1.3.8.7
1.3.8.1
1.3.8.5
1.3.99.-
3-Hydroxy-isovaleryl-CoA
4.2.1.17
3-Methylbut-2-enoyl-CoA
Methacrylyl-CoA
trans-2-Methyl-but-2-enoyl-CoA
3-Hydroxyisovalerate
6.4.1.4
3-Methyl-glutaconyl-CoA
4.2.1.18
(S)-3-Hydroxy-3-methylglutaryl-CoA
(S)-3-Hydroxy-isobutyryl-CoA
(S)-3-Hydroxy-2-methylbutyryl-CoA
3.1.2.4
1.1.1.35
1.1.1.178
Terpenoid backbone biosynthesis
Acetoacetate
4.1.3.4
(S)-3-Hydroxy-isobutyrate
2-Methyl-acetoacetyl-CoA
2.3.3.10
2.8.3.5
6.2.1.16
Acetyl-CoA
1.1.1.31
2.3.1.16
2.3.1.9
Acetoacetyl-CoA
Methylmalonate semialdehyde
Propanoyl-CoA
(S)-3-Amino-isobutanoate
2.6.1.18
2.6.1.22
1.2.1.27
(R)-3-Amino-isobutanoate
2.6.1.40
Propanoate metabolism
1.2.1.3
1.2.3.1
6.4.1.3
Citrate cycle
Pyrimidine metabolism
3.1.2.17
Methylmalonate
6.2.1.-
(S)-Methyl-malonyl-CoA
Succinyl-CoA
5.4.99.2
5.1.99.1
(R)-Methylmalonyl-CoA
Biosynthesis of 12-, 14- and 16-membered macrolides
Biosynthesis of type II polyketide backbone
00280 12/16/22
(c) Kanehisa Laboratories

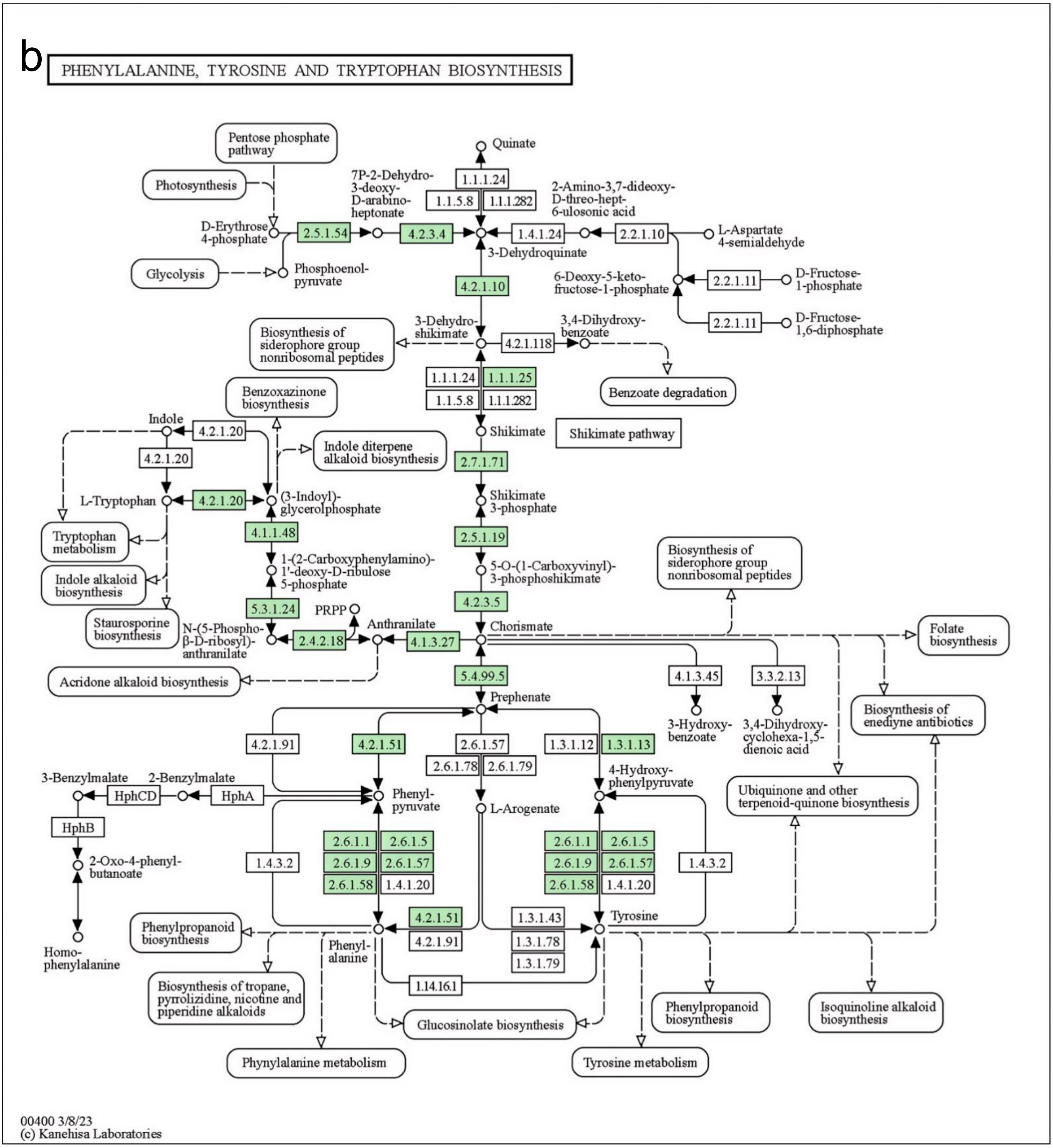

b
PHENYLALANINE, TYROSINE AND TRYPTOPHAN BIOSYNTHESIS
Pentose phosphate pathway
Photosynthesis
Quinate
1.1.1.24
1.1.5.8
1.1.1.282
7P-2-Dehydro-3-deoxy-D-arabino-heptonate
2-Amino-3,7-dideoxy-D-threo-hept-6-ulosonic acid
D-Erythrose 4-phosphate
2.5.1.54
4.2.3.4
1.4.1.24
2.2.1.10
L-Aspartate 4-semialdehyde
3-Dehydroquinate
Glycolysis
Phosphoenol-pyruvate
4.2.1.10
6-Deoxy-5-keto-fructose-1-phosphate
2.2.1.11
D-Fructose-1-phosphate
D-Fructose-1,6-diphosphate
3-Dehydro-shikimate
3,4-Dihydroxy-benzoate
Biosynthesis of siderophore group nonribosomal peptides
4.2.1.118
Benzoate degradation
1.1.1.25
Benzoxazinone biosynthesis
Indole
4.2.1.20
Shikimate
Shikimate pathway
Indole diterpene alkaloid biosynthesis
2.7.1.71
L-Tryptophan
(3-Indoyl)-glycerolphosphate
Shikimate 3-phosphate
Tryptophan metabolism
4.1.1.48
2.5.1.19
1-(2-Carboxyphenylamino)-1'-deoxy-D-ribulose 5-phosphate
5-O-(1-Carboxyvinyl)-3-phosphoshikimate
Indole alkaloid biosynthesis
5.3.1.24
PRPP
4.2.3.5
Staurosporine biosynthesis
N-(5-Phospho-β-D-ribosyl)-anthranilate
2.4.2.18
Anthranilate
4.1.3.27
Chorismate
Folate biosynthesis
Acridone alkaloid biosynthesis
5.4.99.5
4.1.3.45
3.3.2.13
Prephenate
3-Hydroxy-benzoate
3,4-Dihydroxy-cyclohexa-1,5-dienoic acid
Biosynthesis of enediyne antibiotics
4.2.1.91
4.2.1.51
2.6.1.57
1.3.1.12
1.3.1.13
2.6.1.78
2.6.1.79
3-Benzylmalate
2-Benzylmalate
HphCD
HphA
Phenyl-pyruvate
L-Arogenate
4-Hydroxy-phenylpyruvate
Ubiquinone and other terpenoid-quinone biosynthesis
HphB
2-Oxo-4-phenyl-butanoate
1.4.3.2
2.6.1.1
2.6.1.5
2.6.1.9
2.6.1.58
1.4.1.20
Phenylpropanoid biosynthesis
1.3.1.43
Tyrosine
Homo-phenylalanine
Phenyl-alanine
1.3.1.78
1.3.1.79
Biosynthesis of tropane, pyrrolizidine, nicotine and piperidine alkaloids
1.14.16.1
Glucosinolate biosynthesis
Isoquinoline alkaloid biosynthesis
Phynylalanine metabolism
Tyrosine metabolism
00400 3/8/23
(c) Kanehisa Laboratories

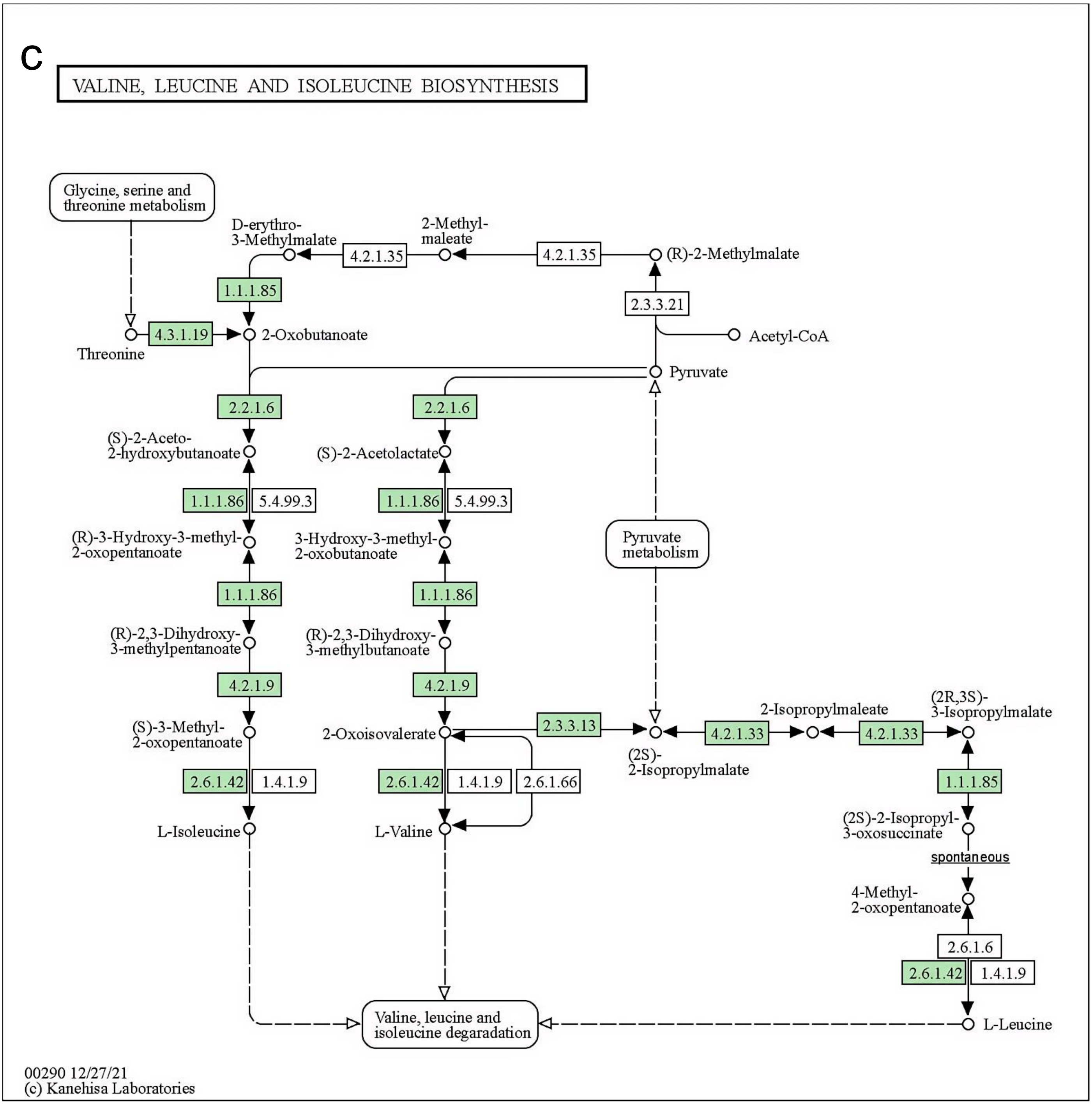
C
VALINE, LEUCINE AND ISOLEUCINE BIOSYNTHESIS
Glycine, serine and threonine metabolism
D-erythro-3-Methylmalate
2-Methyl-maleate
4.2.1.35
4.2.1.35
(R)-2-Methylmalate
2.3.3.21
Acetyl-CoA
1.1.1.85
Threonine
4.3.1.19
2-Oxobutanoate
Pyruvate
2.2.1.6
2.2.1.6
(S)-2-Aceto-2-hydroxybutanoate
(S)-2-Acetolactate
1.1.1.86
5.4.99.3
1.1.1.86
5.4.99.3
(R)-3-Hydroxy-3-methyl-2-oxopentanoate
3-Hydroxy-3-methyl-2-oxobutanoate
Pyruvate metabolism
1.1.1.86
1.1.1.86
(R)-2,3-Dihydroxy-3-methylpentanoate
(R)-2,3-Dihydroxy-3-methylbutanoate
4.2.1.9
4.2.1.9
(S)-3-Methyl-2-oxopentanoate
2-Oxoisovalerate
2.3.3.13
(2S)-2-Isopropylmalate
4.2.1.33
2-Isopropylmaleate
4.2.1.33
(2R,3S)-3-Isopropylmalate
2.6.1.42
1.4.1.9
2.6.1.42
1.4.1.9
2.6.1.66
1.1.1.85
L-Isoleucine
L-Valine
(2S)-2-Isopropyl-3-oxosuccinate
spontaneous
4-Methyl-2-oxopentanoate
2.6.1.6
2.6.1.42
1.4.1.9
Valine, leucine and isoleucine degaradation
L-Leucine
00290 12/27/21
(c) Kanehisa Laboratories

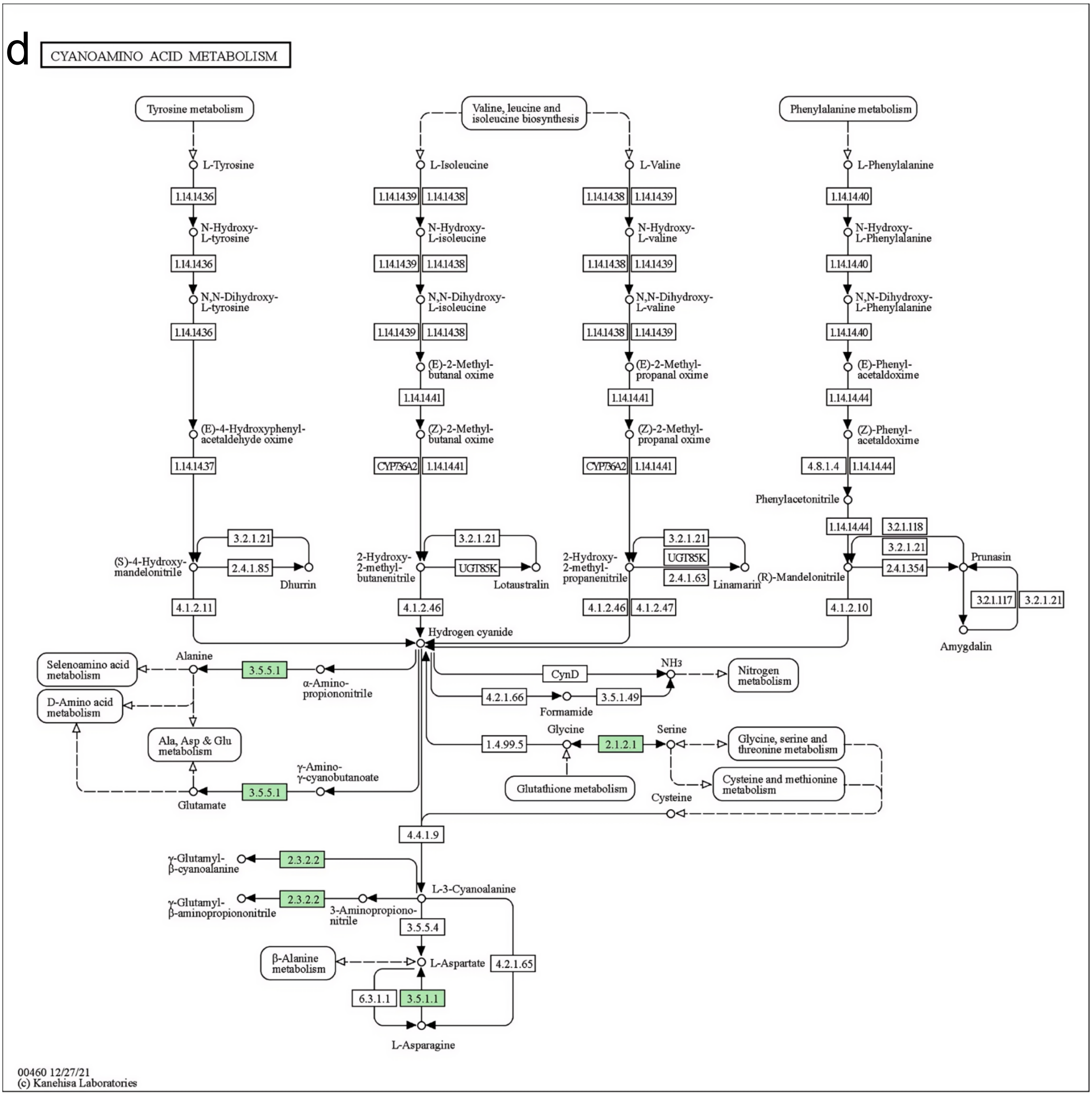
d
CYANOAMINO ACID METABOLISM
Tyrosine metabolism
Valine, leucine and isoleucine biosynthesis
Phenylalanine metabolism
L-Tyrosine
L-Isoleucine
L-Valine
L-Phenylalanine
1.14.14.36
1.14.14.39
1.14.14.38
1.14.14.40
N-Hydroxy-L-tyrosine
N-Hydroxy-L-isoleucine
N-Hydroxy-L-valine
N-Hydroxy-L-Phenylalanine
N,N-Dihydroxy-L-tyrosine
N,N-Dihydroxy-L-isoleucine
N,N-Dihydroxy-L-valine
N,N-Dihydroxy-L-Phenylalanine
(E)-2-Methyl-butanal oxime
(E)-2-Methyl-propanal oxime
(E)-Phenyl-acetaldoxime
1.14.14.41
1.14.14.44
(E)-4-Hydroxyphenyl-acetaldehyde oxime
(Z)-2-Methyl-butanal oxime
(Z)-2-Methyl-propanal oxime
(Z)-Phenyl-acetaldoxime
1.14.14.37
CYP736A2
4.8.1.4
Phenylacetonitrile
3.2.1.21
3.2.1.118
(S)-4-Hydroxy-mandelonitrile
2.4.1.85
Dhurrin
2-Hydroxy-2-methyl-butanenitrile
UGT85K
Lotaustralin
2-Hydroxy-2-methyl-propanenitrile
2.4.1.63
Linamarin
(R)-Mandelonitrile
2.4.1.354
Prunasin
3.2.1.117
Amygdalin
4.1.2.11
4.1.2.46
4.1.2.47
4.1.2.10
Hydrogen cyanide
Selenoamino acid metabolism
Alanine
3.5.5.1
α-Amino-propiononitrile
CynD
NH3
Nitrogen metabolism
D-Amino acid metabolism
4.2.1.66
Formamide
3.5.1.49
Ala, Asp & Glu metabolism
Glycine
1.4.99.5
2.1.2.1
Serine
Glycine, serine and threonine metabolism
γ-Amino-γ-cyanobutanoate
Glutathione metabolism
Cysteine and methionine metabolism
Glutamate
Cysteine
4.4.1.9
γ-Glutamyl-β-cyanoalanine
2.3.2.2
L-3-Cyanoalanine
γ-Glutamyl-β-aminopropiononitrile
3-Aminopropiono-nitrile
3.5.5.4
β-Alanine metabolism
L-Aspartate
4.2.1.65
6.3.1.1
3.5.1.1
L-Asparagine
00460 12/27/21
(c) Kanehisa Laboratories

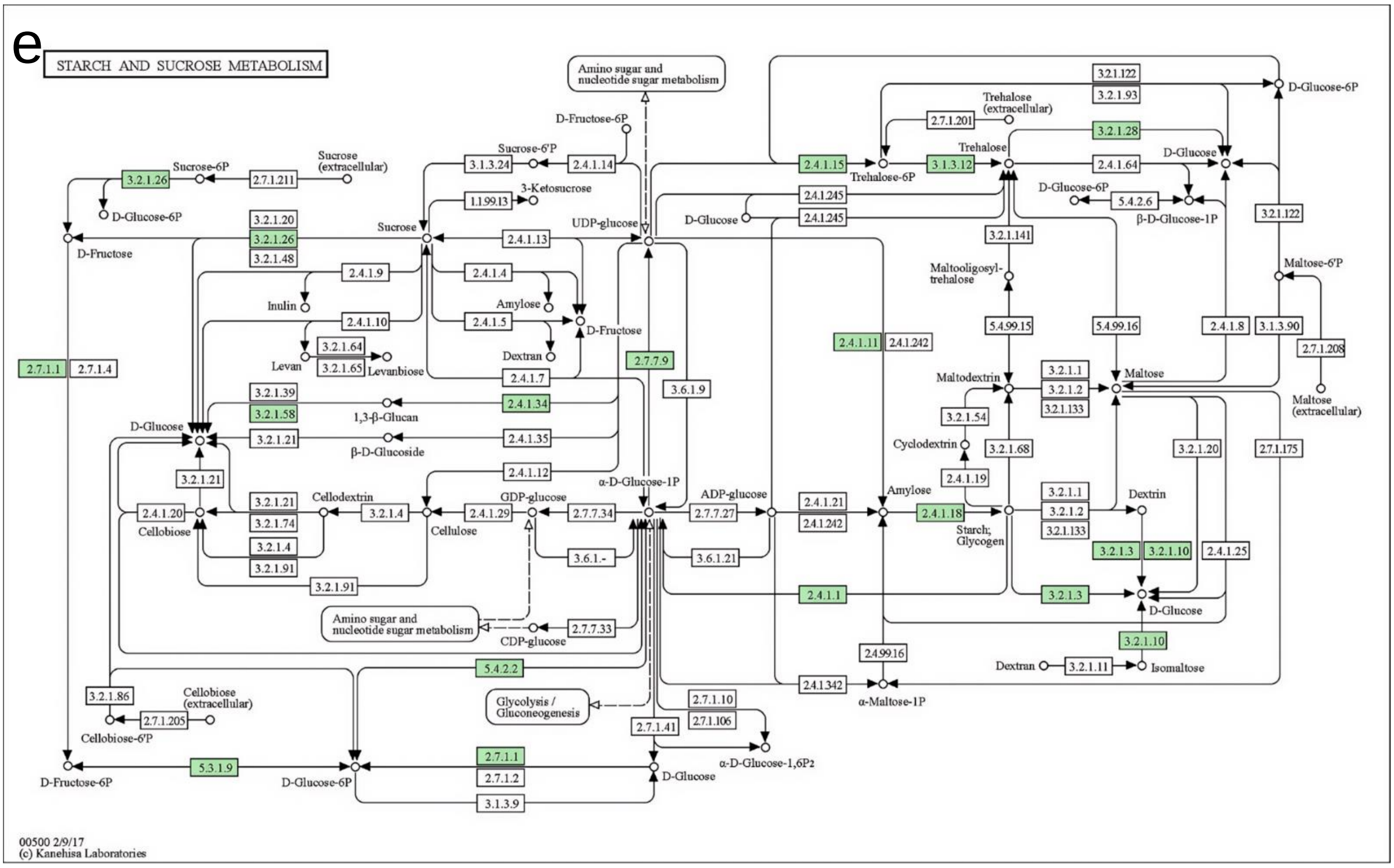
e
STARCH AND SUCROSE METABOLISM
Amino sugar and nucleotide sugar metabolism
D-Fructose-6P
Sucrose-6'P
3.1.3.24
2.4.1.14
Sucrose-6P
3.2.1.26
2.7.1.211
Sucrose (extracellular)
D-Glucose-6P
1.1.99.13
3-Ketosucrose
3.2.1.20
3.2.1.26
3.2.1.48
Sucrose
2.4.1.13
UDP-glucose
D-Fructose
2.4.1.9
2.4.1.4
Inulin
Amylose
2.4.1.10
2.4.1.5
D-Fructose
3.2.1.64
3.2.1.65
Levan
Levanbiose
Dextran
2.7.1.1
2.7.1.4
2.4.1.7
2.7.7.9
3.6.1.9
3.2.1.39
3.2.1.58
2.4.1.34
1,3-β-Glucan
D-Glucose
3.2.1.21
2.4.1.35
β-D-Glucoside
3.2.1.21
2.4.1.12
α-D-Glucose-1P
2.4.1.20
3.2.1.21
3.2.1.74
Cellodextrin
3.2.1.4
Cellobiose
Cellulose
GDP-glucose
2.4.1.29
2.7.7.34
ADP-glucose
2.7.7.27
3.2.1.4
3.2.1.91
3.6.1.-
3.6.1.21
3.2.1.91
Amino sugar and nucleotide sugar metabolism
CDP-glucose
2.7.7.33
5.4.2.2
3.2.1.86
Cellobiose (extracellular)
2.7.1.205
Cellobiose-6'P
Glycolysis / Gluconeogenesis
2.7.1.10
2.7.1.106
2.7.1.41
α-D-Glucose-1,6P2
D-Fructose-6P
5.3.1.9
D-Glucose-6P
2.7.1.1
2.7.1.2
D-Glucose
3.1.3.9
3.2.1.122
3.2.1.93
D-Glucose-6P
2.7.1.201
Trehalose (extracellular)
3.2.1.28
2.4.1.15
3.1.3.12
Trehalose
Trehalose-6P
2.4.1.64
D-Glucose
2.4.1.245
2.4.1.245
D-Glucose
D-Glucose-6P
5.4.2.6
β-D-Glucose-1P
3.2.1.122
3.2.1.141
Maltooligosyl-trehalose
Maltose-6'P
2.4.1.11
2.4.1.242
5.4.99.15
5.4.99.16
2.4.1.8
3.1.3.90
2.7.1.208
Maltodextrin
3.2.1.1
3.2.1.2
3.2.1.133
Maltose
Maltose (extracellular)
3.2.1.54
Cyclodextrin
3.2.1.68
3.2.1.20
2.7.1.175
2.4.1.19
2.4.1.21
2.4.1.242
Amylose
2.4.1.18
Starch; Glycogen
3.2.1.1
3.2.1.2
3.2.1.133
Dextrin
3.2.1.3
3.2.1.10
2.4.1.25
2.4.1.1
3.2.1.3
D-Glucose
2.4.99.16
3.2.1.10
Dextran
3.2.1.11
Isomaltose
2.4.1.342
α-Maltose-1P
00500 2/9/17
(c) Kanehisa Laboratories

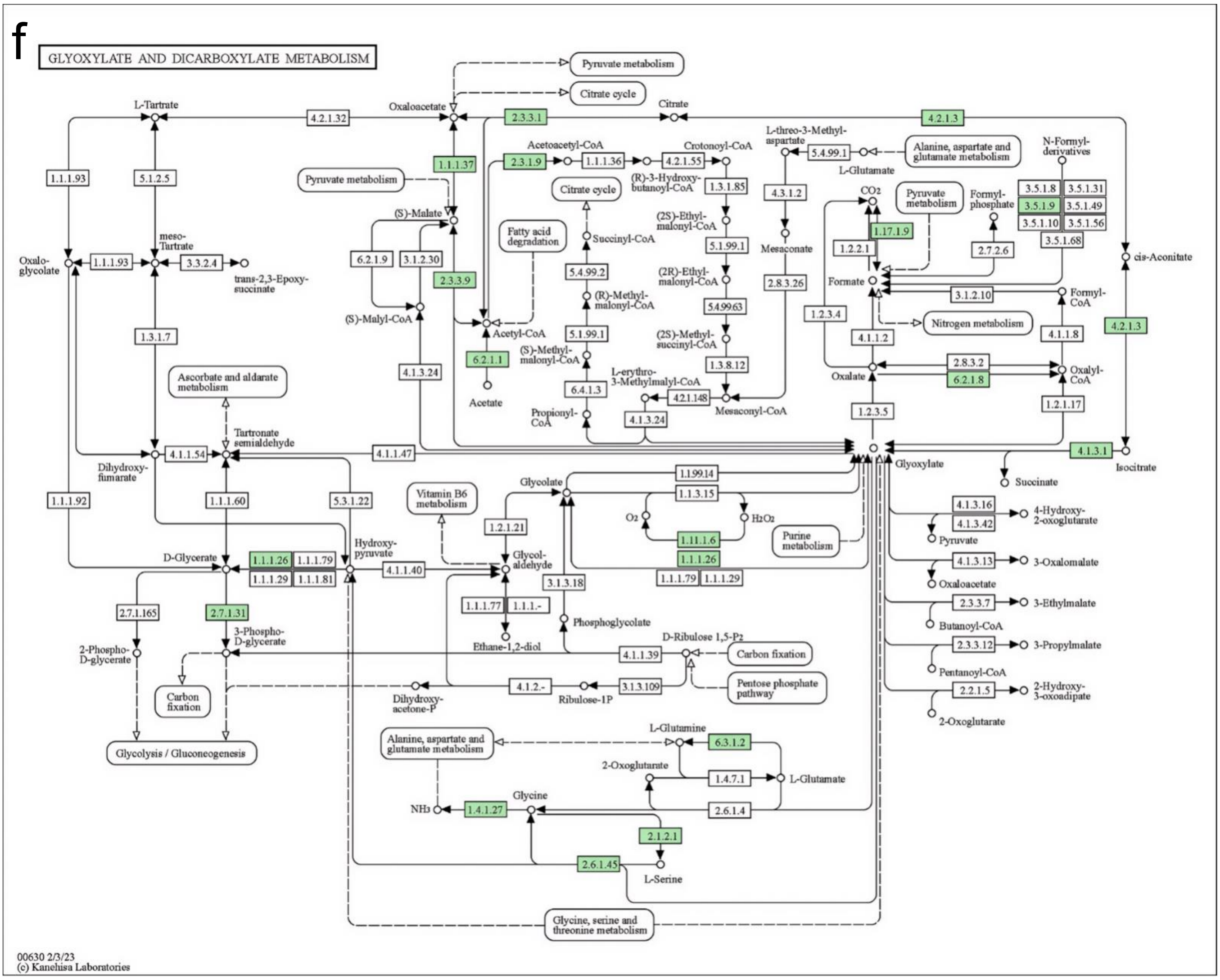

Figure S4. Major KEGG pathway maps of identified metabolites of *B. roseoflavus* and *L. asiatica* at different drying methods, namely, a) valine, leucine and isoleucine degradation, b) phenylalanine, tyrosine and tryptophan biosynthesis, c) valine, leucine and isoleucine biosynthesis, d) cyanoamino acid metabolism, e) starch and sucrose metabolism, f) glyoxylate and dicarboxylate metabolism.
